# Linguistic emergence from a networks approach: The case of modern Chinese two-character words

**DOI:** 10.1371/journal.pone.0259818

**Published:** 2021-11-11

**Authors:** Jin Cong, Haitao Liu

**Affiliations:** 1 School of Foreign Languages, Ludong University, Yantai, China; 2 Department of Linguistics, Zhejiang University, Hangzhou, China; 3 Institute of Quantitative Linguistics, Beijing Language and Culture University, Beijing, China; University of Sao Paulo, BRAZIL

## Abstract

The models of linguistic networks and their analytical tools constitute a potential methodology for investigating the formation of structural patterns in actual language use. Research with this methodology has just started, which can hopefully shed light on the emergent nature of linguistic structure. This study attempts to employ linguistic networks to investigate the formation of modern Chinese two-character words (as structural units based on the chunking of their component characters) in the actual use of modern Chinese, which manifests itself as continuous streams of Chinese characters. Network models were constructed based on authentic Chinese language data, with Chinese characters as nodes, their co-occurrence relations as directed links, and the co-occurrence frequencies as link weights. Quantitative analysis of the network models has shown that a Chinese two-character word can highlight itself as a two-node island, i.e., a cohesive sub-network with its two component characters co-occurring more frequently than they co-occur with the other characters. This highlighting mechanism may play a vital role in the formation and acquisition of two-character words in actual language use. Moreover, this mechanism may also throw some light on the emergence of other structural phenomena (with the chunking of specific linguistic units as their basis).

## 1. Introduction

The actual use of language has attracted increasing research interest in recent decades, especially regarding its role in the formation, acquisition, evolution, etc., of linguistic structure. Viewed from a usage-based perspective, linguistic structure emerges from language experience based on exemplars of language use to which language users are exposed [1, 2].

Although the structural patterns generally lack natural boundaries in actual language use, they can establish themselves and can be extracted by language users by standing out through particular relational patterns (e.g., strong associations between their component units). This has been supported, directly or indirectly, by various studies [3–5], which suggest that the quantitative patterns of actual language use may shed light on the emergent nature of linguistic structure.

In order for the quantitative patterns of actual language use to shed light on linguistic emergence, two prerequisites need to be considered, namely, appropriate modeling and quantitative measures of actual language use.

The notion of networks often finds its way into the modeling of language experience. Hudson [6], for instance, held that language is represented as part of the conceptual network of human knowledge and employed the notion of networks in structural analysis at various language levels. Bybee [7] argued that language experience can be conceived of as networks composed of linguistic units (e.g., words) and their relations in actual language use, such as associations (e.g., phonetic and semantic similarities) and co-occurrence relations. Furthermore, Bybee [7] also sought a network-based explanation of how particular structural patterns (e.g., idioms and morphological patterns) emerge from language experience. The notion of networks constitutes a relational approach to language in that anything (be it a linguistic unit, a pattern of use, or a linguistic feature) is considered against a larger context of linguistic units and their relations. This marks an intrinsic advantage of networks thinking in usage-based inquiries into linguistic structure, for both linguistic structure and the context of actual language use manifest themselves as linguistic units and their relations.

Linguistic structure is held to emerge from the co-occurrence relations of linguistic units in actual language use [2, 7]. Linguists have available to them a wide range of measures for co-occurrence strength [8], with varying computational costs. Simple measures (e.g., frequency and probability) are adopted from time to time in usage-based linguistics [7], largely due to their close bearing on language experience and thus the mechanisms of linguistic emergence. However, the mere inspection of these simple measures alone might still be insufficient for a systematic understanding of the formation of structural patterns in actual language use. For instance, a high co-occurrence frequency of two units does not necessarily mean a meaningful unit, and vice versa. However, the advantages of simple measures are worth considering. The specific roles of these measures in linguistic emergence might be further understood with appropriate models and analytical tools of actual language use. A networks approach may contribute to this understanding by taking into account the larger relational background of any co-occurrence relation and its strength.

To recap, network models plus simple measures for co-occurrence strength (such as frequency) may constitute an excellent approach to the emergence of linguistic structure. However, such networks thinking needs to be converted into substantial studies.

Rooted in a multi-disciplinary background, the models and quantitative tools of linguistic networks [9, 10] constitute an operational methodology for networks thinking in linguistics. The basic form of a linguistic network model *N* can be generally represented as *N = (V*, *E)*, with *V* being the set of *nodes* (vertices) and *E* the set of *links* (edges). Various language levels as language sub-systems can be modeled and analyzed quantitatively as linguistic networks, with the corresponding linguistic units (e.g., words) as nodes and their relations (e.g., structural and semantic relations between the words) of a particular type as links. The models can also include extra information concerning the features of linguistic units and their relations (such as co-occurrence frequencies of linguistic units) if necessary. Structural analysis of linguistic networks is supported by a wide range of quantitative tools, which rely on relational data [11] in that they always focus on the relations between linguistic units and features. These tools can hopefully shed light on structural patterns (as relational phenomena) of language at different scales of granularity.

Earlier inquiries into linguistic networks have helped to appreciate the system-level complexity of particular language sub-systems [12, 13]. It has been shown later that such an understanding of the system-level complexity of language can also shed light on language development [14], special education [15], language typology [16], and so forth. In practical terms, such an understanding can contribute to various NLP tasks [17–19]. More recent studies have extended the scope of research. New ways of modeling have been adopted. For instance, the analysis of linguistic networks of different sub-systems of the same language has deepened the understanding of language as a multi-level system [20, 21]. The use of multi-layer sentence networks (distinguishing links between sentences from the same document and those connecting sentences from different documents) has facilitated NLP undertakings such as multi-document summarization [22]. Moreover, increasing interest has been devoted to the rather microscopic features of linguistic networks, which are examined with measures including those concerning node centrality [22–24].

Inquiries into the less macroscopic features (e.g., the various cohesive sub-networks) of linguistic networks can hopefully shed light on how structural linguistic units are formed by their component units, which in turn may contribute to a usage-based understanding of linguistic emergentism. This line of research has just started. For instance, Goh et al. [25] have shown that the motifs (cohesive sub-networks) in word co-occurrence networks based on authentic English texts function as network shortcuts, and the motif densities are drastically lowered when the texts are shuffled. More importantly, their results have revealed a link between these network motifs and linguistic constructions, which points to the mechanism of these constructions’ emergence from the patterns of actual language use.

The formation of Chinese words (as structural units based on the chunking of their component characters) in actual language use constitutes an interesting case study in this line of research. This study explores the formation of Chinese two-character words (henceforth, CTCWs) in the actual use of modern Chinese, which manifests itself as continuous streams of Chinese characters and which was modeled as character co-occurrence networks. The network models consist of Chinese characters as nodes, their co-occurrence relations as directed links, and the co-occurrence frequencies as link weights. Quantitative analysis of the network models has shown that a Chinese two-character word can highlight itself as a two-node island, i.e., a cohesive sub-network with its two component characters co-occurring more frequently than they co-occur with the other characters. This highlighting mechanism may play a vital role in the formation and acquisition of CTCWs in actual language use. Moreover, this mechanism may also throw some light on the emergence of other structural phenomena (with the chunking of specific linguistic units as their basis).

## 2. Chinese words as structural units

Unlike other languages such as English, Chinese words lack natural boundaries in actual language use. Morphological markers such as affixes, which can serve as word boundaries, are generally non-existent in Chinese [26: 46]. Moreover, even in written Chinese, the words are not explicitly separated. In other words, they are not separated by spaces as in the case of English (see Example 1 for illustration). The actual use of Chinese language manifests itself as continuous streams of characters, which are generally understood as morphemes of the language [27], the elements of word formation. Each character is a linguistic unit corresponding to one syllable, one orthographic symbol, and usually a particular amount of meaning. To recap, spoken Chinese and written Chinese can be seen as continuous streams of syllables and orthographic symbols, respectively. Each Chinese word is a structural unit, consisting of one, two, or sometimes even more characters chunked together. CTCWs constitute a dominant portion of modern Chinese vocabulary.

Example 1 below illustrates how the actual use of modern Chinese looks like, especially the non-existence of spaces between words. It is an excerpt from the Chinese version of Bloomfield’s *Language* [28: 21], which is followed by its English original.

### Example 1

至于语言变化问题, 我们已有足够的事实可以证明, 所有的语言都同样有变化过程, 而且都倾向于同一方向。甚至很特殊的变化类型, 在差别最大语言里也可以发生非常相同的变化, 只不过是独立地进行而已。

As to change in language, we have enough data to show that the general processes of change are the same in all languages and tend in the same direction. Even very specific types of change occur in much the same way, but independently, in the most diverse languages.

Example 1 shows a stream of 85 Chinese characters (tokens), separated only by 7 punctuation marks. Example 2 below shows one clause in Example 1 with all the words segmented by spaces (1^st^ line), its phonetic transcription using the official romanization system for Standard Chinese (2^nd^ line), word-by-word gloss in English (3^rd^ line, REL = relative marker), and the English original (4^th^ line).

### Example 2

所有 的 语言 都 同样 有 变化 过程

suǒyǒu de yǔyán dōu tóngyàng yǒu biànhuà guòchéng

all REL language all same have change process

[T] he general processes of change are the same in all languages…

In actual language use, any two co-occurring (i.e., adjacent) Chinese characters may fall into either of the two cases: (1) they are within the same word (e.g., any two characters which are not separated by a space in Example 2, such as 语 (yǔ) and 言 (yán)); (2) they constitute the boundary between two adjacent words (e.g., any two characters separated by a space in Example 2, such as 的 (de) and 语 (yǔ)), i.e., the two characters belong to two different words but happen to be adjacent.

CTCWs constitute an interesting subject of inquiries into the formation of structural patterns from a usage-based perspective. Although it has proved possible to segment Chinese words largely on the basis of statistical features of the character streams [29, 30], the statistics adopted by these engineering attempts generally involve complicated computation, which may be rather removed from how the patterns of actual language use affect language experience. In this study, priority is given to rather simple measures of actual language use (especially frequency), which are examined from a networks approach.

## 3. A networks approach to frequency

Frequency is held to play a fundamental role in the formation, evolution, learning, and mental representation of structural patterns at various language levels [5, 31]. For instance, the frequencies of linguistic units and their combinations have a profound impact on the way how language is chunked in memory, how such chunks are connected, and how easily they are accessed [1].

Viewed from a usage-based perspective, Chinese words emerge from the co-occurrence relations of Chinese characters in actual language use. Unless otherwise specified, co-occurrence in this paper means that two linguistic units (e.g., Chinese characters) are immediate neighbors of each other and thus form a bigram as an ordered pair. Given two linguistic units *u* and *v*, the two ordered pairs they form, *uv* and *vu*, are different co-occurrence relations or bigrams. The three co-occurrence relations of characters (or character bigrams) at the beginning of Example 1, for instance, are 至于 (zhìyú), 于语 (yúyǔ), and 语言 (yǔyán). In actual language use, any CTCW can be seen as a reusable character bigram denoting one or more meanings. This repetition of co-occurrence helps to entrench the character bigram as a structural whole. Co-occurrence frequencies of characters, therefore, may play a crucial role in the formation and acquisition of CTCWs in actual language use. For instance, the top 10 most frequent character bigrams in the Lancaster Corpus of Modern Chinese [32] are all CTCWs.

However, a more in-depth understanding of the role of frequency (e.g., the specific scope of its effects) is needed. A general law of human language is that word frequency varies drastically [33]: only few of the words being highly frequent while the majority of them rather infrequent. The same is true of CTCWs. A large number of character bigrams with low frequencies may still be CTCWs. What is worse is that many non-word character bigrams exhibit rather high frequency levels. The frequency level alone, therefore, may not be a reliable indicator of whether a character bigram forms a word or not. However, the actual language use to which language users are exposed is ever-growing and ever-changing [7]. Frequency may always play a role in the formation of structural patterns, for even the least frequently-used units or patterns according to corpus statistics can occur repeatedly in actual language use. In order for a word *uv* to stand out as a cohesive unit in actual language use, the frequency of *uv* may not have to compete with all the other character bigrams in the entire context. Instead, it may be more plausible to examine the frequency of *uv* against its immediate context, i.e., the bigrams which *u* and *v* respectively form with other characters.

**Fig 1:**
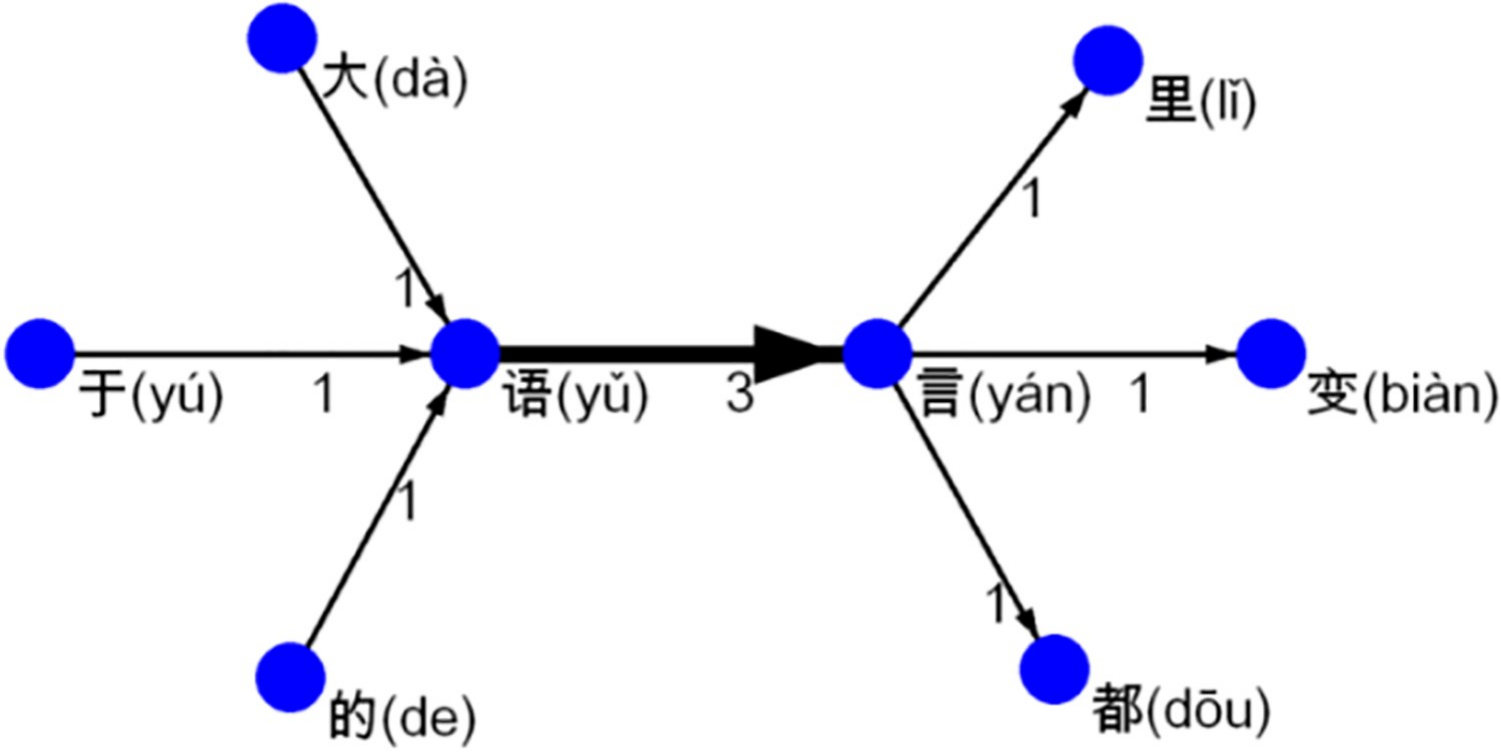
A network model of 语言 (yǔyán, ‘language’) with its immediate context in the text of Example 1.

In authentic Chinese language use, any two characters *u* and *v* forming a bigram *uv* also co-occur with other characters at the same time, and these co-occurrence relations constitute the immediate context of bigram *uv*. In Example 1, 语 (yǔ) and 言 (yán), in addition to co-occur with each other, also co-occur respectively with other characters, hence the immediate context of 语言. The bigram 语言 (a CTCW meaning ‘language’) and its immediate context can be modeled as a network in Fig 1, whereby any two characters forming a bigram in the text is connected by a directed link (an arrowed line indicating the order of characters), and the frequency of each bigram is represented by a value attached to the corresponding link and the thickness of the link.

As discussed earlier, a character bigram constitutes either a word (or part of it) or the boundary between two adjacent words. In the former case, the two characters may form a CTCW (e.g., 语言 in Example 1) and thus can be used repeatedly. A character bigram forming the boundary between two words, on the other hand, is due to the co-occurring of the two words as a result of syntactic arrangement of the utterance. Considering the flexibility of syntax, such a bigram (e.g., those formed by 语 or 言 with the other six characters as illustrated by Fig 1) may not exhibit the same degree of reusability as a CTCW.

It follows that if two characters form a CTCW, they may tend to co-occur with each other more frequently than they co-occur with other characters. Given a character bigram *uv* found in a corpus, its own frequency can be seen as its internal tightness, and the frequencies *u* and *v* co-occur respectively with their other immediate neighbors can be seen as the external tightness of *uv*. A CTCW, therefore, is likely to exhibit itself as a two-character chunk with stronger internal than external tightness. In this way, a CTCW as a cohesive structural whole can stand out in its immediate context, which may contribute abundantly to its emergence from actual language use. In Example 1, for instance, 语言as a CTCW stands out as such a chunk (see Fig 1).

In sum, a networks approach to co-occurrence frequency may be more plausible in accounting for the emergence of a CTCW, which focuses on the frequency level of the corresponding character bigram relative to those of the other bigrams in its immediate context, instead of its frequency level alone. Such an approach can be operationalized using network analysis of authentic Chinese language data.

## 4. Materials and methods

In order to understand the emergence of CTCWs, this study attempts to examine (1) the two-character chunks with stronger internal tightness as previously defined, and (2) how such chunks relate to CTCWs. Network analysis of authentic Chinese language data can be employed to detect such chunks with stronger internal tightness.

### 4.1 Materials

The authentic language data adopted in this study are from two corpora, namely, the Lancaster Modern Chinese Corpus (LCMC) [32] and the Leiden Weibo Corpus (LWC) [34], which cover the formal and informal use of modern Chinese, respectively.

LCMC is a balanced corpus of modern written Chinese with about 1.6 million Chinese character tokens (roughly 1 million word tokens). It consists of 500 samples, 3,200 Chinese character tokens apiece, and covers 15 text categories. Two sub-corpora of LCMC were adopted for this study, namely, those of press reportage (LCMC_A, about 120,000 character tokens) and science (academic prose) (LCMC_J, about 230,000 character tokens). These corresponds to two representative genres of formal language use of Chinese.

LWC consists of over 5,100,000 messages posted on Weibo, China’s leading social media platform. Compared with the language data in LCMC, those in LWC are rather close to daily spoken interactions. In this study, the first 8,000 messages with substantial linguistic content (i.e., consisting of at least one word) in LWC were selected as a sub-corpus (about 250,000 character tokens) concerning the informal use of Chinese.

### 4.2 Network modeling

Considering the advantages of linguistic networks in dealing with relational phenomena (such as linguistic structure), it is necessary to construct and analyze linguistic network models based on authentic language data. These networks have linguistic units as nodes and their relations (e.g., co-occurrence, syntactic dependency, or semantic dependency relations) observed in actual language use as links. Network models constructed in this way, therefore, are potentially capable of modeling language users’ exemplar-based language experience. Quantitative analysis of such networks can help to detect various cohesive clusters formed by linguistic units in actual language use, which can hopefully shed empirical light on the emergent mechanisms of particular structural patterns, such as CTCWs.

As previously noted, the actual use of Chinese language can be seen as continuous streams of characters (as the immediately-observable linguistic units). In network analysis, these can be modeled by directed and weighted character co-occurrence networks. Such a network can be represented as *N = (V*, *E*, *w)*, with *V* being the set of all the characters (types) in the corpus, *E* the set of co-occurrence relations of characters, and *w* the set of link weights representing frequency values of the corresponding co-occurrence relations. Only Chinese characters were counted as network nodes. Others symbols, such as Arabic numerals and punctuation marks, were all ruled out. Any two co-occurring characters *u* and *v* forming a bigram *uv* were connected by a directed link pointing from *u* to *v* in the corresponding network. In rare cases, a character *u* might co-occur with itself to form a bigram *uu*. For instance, some CTCWs are formed by reduplicating the same character, such as 叔叔 (shūshu, ‘uncle’), 狒狒 (fèifèi, ‘baboon’), and 天天 (tiāntiān, ‘every day’). A co-occurrence relation of this type was represented by a link pointing from one character to itself, which is termed as a *self-loop*. Note that two characters separated by a punctuation mark representing a pause (such as comma, and period 。) did not count as co-occurring. The frequency of any co-occurrence relation of characters is the weight of the corresponding link.

**Fig 2:**
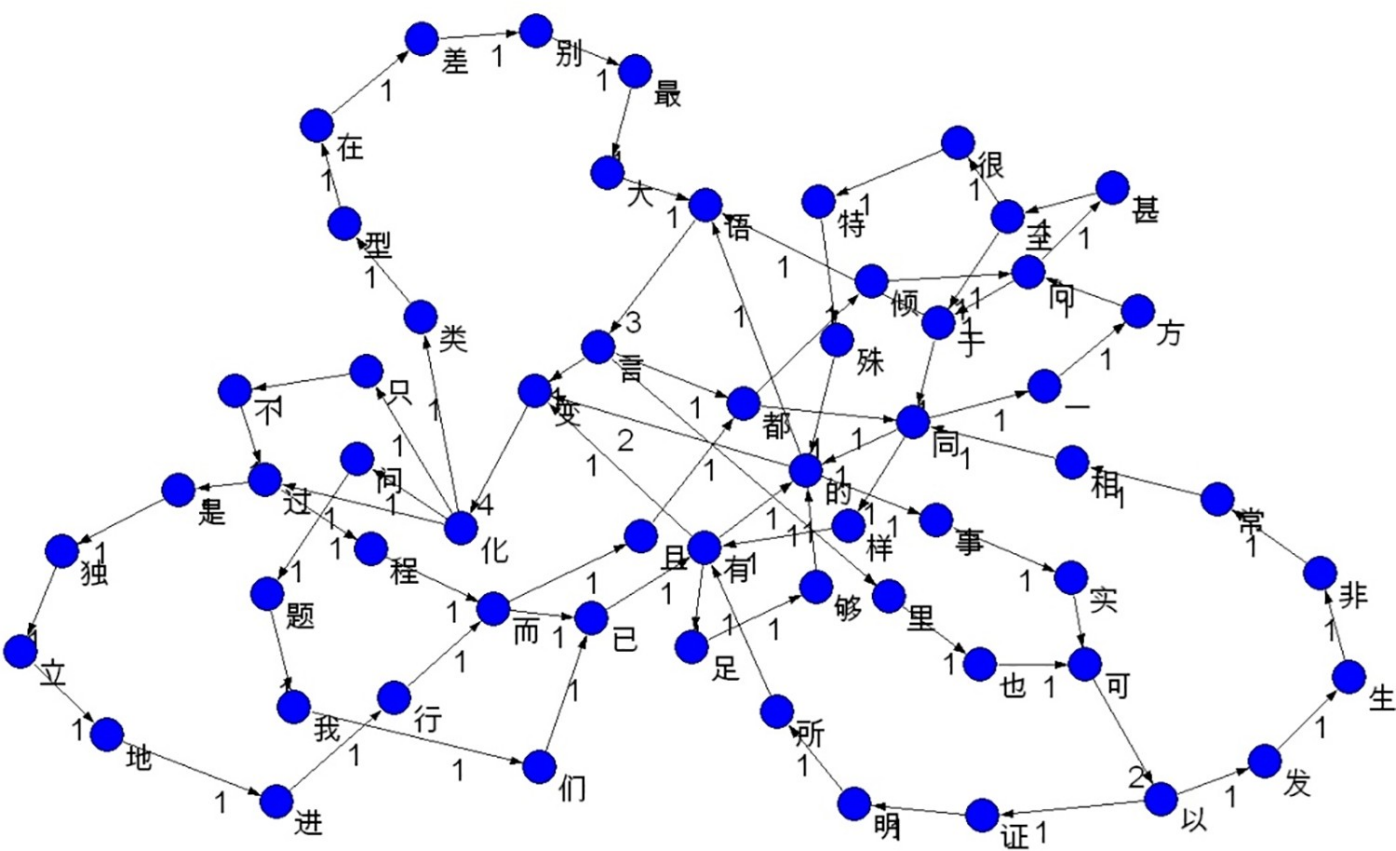
A linguistic network based on the text of Example 1.

Fig 2 displays the linguistic network constructed based on the text of Example 1, with the scheme described above. The network in Fig 1 is a sub-network of it.

In Fig 2, the number attached to each directed link represents its weight, that is, the frequency of the corresponding bigram. For instance, as the character bigram (in fact a CTCW) 语言 (yǔyán, ‘language’) occurred 3 times in the text, the weight of the corresponding link is 3 (see also Fig 1).

With the scheme introduced above, the three sub-corpora were converted into three network models (henceforth, Networks LCMC_A, LCMC_J, and LWC), respectively.

### 4.3 Data analysis

As previously discussed, in a body of authentic language data, if two characters co-occur more frequently than they co-occur with the other characters, they form a chunk with stronger internal than external tightness in its immediate context. A chunk of this type is in line with the notion of *islands* [35] in network analysis. An island is defined as a sub-network, whose inner-links, those connecting its member nodes, all have greater weight values than its outer-links, those connecting its member nodes with nodes outside it. A two-character chunk with stronger internal tightness due to its repeated use (e.g., 语言 (yǔyán, ‘language’) as illustrated by Fig 1), therefore, corresponds to a two-node island (henceforth, TNI) in a linguistic network constructed as previously described.

The classical definition of islands is based on networks without self-loops. In the actual use of Chinese, the co-occurring characters do form self-loops, which are rare yet worth considering. Some of these self-loops are CTCWs, e.g., 叔叔 (shūshu, ‘uncle’). For the purpose of this study, an adjusted definition of TNIs is formulated, so that the self-loops formed by co-occurring characters are considered. Let the directed link between two nodes *u* and *v* be *e* (allowing *u* = *v*, and hence a self-loop), and any other directed link involving *u* or *v e’*. If the weight of *e* is greater than the maximal weight of the latter, i.e., *w(e) > max(w(e’))*, then <*u*, *v*> forms a TNI.

Based on the above definition of TNIs, data analysis of this study took two major steps: (1) extraction of TNIs from the linguistic networks, and (2) determination of the relationship between the TNIs and CTCWs.

Chinese words are rather fluid units in that it is sometimes difficult to draw the boundary between a word and phrase. Dictionaries, lexicons, and lexical databases published officially or academically constitute an operational (though not perfect) tool for wordhood judgment. The sources adopted by this study are: (1) *Contemporary Chinese Dictionary (7*^*th*^ *Edition)* [36] (henceforth, CCD), the most authoritative reference work on modern Chinese words; (2) *Lexicon of Common Words in Contemporary Chinese* [37] (henceforth, LCWCC), released by the State Language Commission of China; (3) the Chinese Lexical Database [38] (henceforth, CLD), a large-scale lexical database for simplified modern Chinese which well reflects the linguistic experience of modern Chinese users; (4) Wiktionary (https://www.wiktionary.org); and (5) Baidu Encyclopedia (https://baike.baidu.com/). Both (4) and (5) are powerful online dictionaries with a good coverage of Chinese neologisms, slang words, and dialect words. Technical terms which are not listed in the above sources were judged on the basis of (1) the terms published by China National Committee for Terms in Sciences and Technologies (partially searched on http://www.termonline.cn/index.htm), and (2) *Dacihai* [39], a large-scale dictionary and encyclopedia of modern Chinese.

In order to ensure the accuracy of wordhood judgment, the TNIs extracted were examined manually based on the above sources and their contexts. A TNI has to meet one major criterion and two minor criteria to be counted as a CTCW. The major criterion is that the TNI should be listed in at least one of the above sources as an entry. The two minor criteria are that (1) not all occurrences of the TNI are boundaries between two adjacent words (i.e., co-occurring but belong to two adjacent words), and (2) not all occurrences of the TNI are parts of longer words. The major criterion is generally sufficient for wordhood judgment, while the two minor criteria help to rule out two very rare cases, which further improves the accuracy of judgment.

## 5. Results

The TNIs extracted from each network were classified into three types: (1) CTCWs, (2) word-like chunks, and (3) non-word chunks. Table 1 displays the number and percentage of TNIs of each type (see Appendices A-C in S1 File). The few TNIs with weight 1 (altogether 3, 1, and 3 in LCMC_A, LCMC_J, and LWC, respectively) have been ruled out, for they were not formed through repeated use of the corresponding character bigrams. Those (altogether 2) in the three sub-networks of Network LCMC_J were treated in the same way.

**Table 1:** Basic information of the TNIs extracted from the three networks.

Network	CTCWs	Word-like chunks	Non-word chunks	Total
LCMC_A	269 (97.46%)	6 (2.17%)	1 (0.36%)	276
LCMA_J	267 (96.74%)	8 (2.90%)	1 (0.36%)	276
LWC	363 (97.58%)	9 (2.42%)	0 (0%)	372

The Type-1 TNIs are those identified as words according to the abovementioned criteria for wordhood judgement. As can be seen from Table 1, CTCWs constitute an overwhelming majority of the TNIs in each network. The CTCWs extracted as TNIs from Network LWC cover some slang words widely used in daily conversations and on social media platforms, such as 粉丝 (fěnsī, ‘fans’), 童鞋 (tóngxié, ‘schoolmate’ or ‘classmate’), and 坑爹 (kēngdiē, ‘cheating’ or ‘deceiving’). These words reflect the informal use of modern Chinese.

The Type-2 TNIs do not count as words according to the criteria of this study. However, they constitute either a two-character phrase (e.g., 万元 (wànyuán, ‘ten thousand Yuan RMB’) or part a word/phrase of three or more characters (e.g., 铃虫 (língchóng) as part of 棉铃虫 (miánlíngchóng, ‘bollworm’) and 红铃虫 (hónglíngchóng, ‘red bollworm’)). As previously noted, the boundary between a two-character phrase and a CTCW is not always clear-cut and even LCWCC and CLD list a number of CTCWs which are sometimes treated as two-character phrases. A two-character phrase behaves very much like a CTCW in that they are both used as a structural and meaningful whole. For instance, 万元 (with 万 (wàn) meaning ‘ten thousand’ and 元 (yuan) ‘Yuan RMB’) can also be seen as a CTCW for a unit of currency.

A Type-3 TNI consists of two characters which respectively belong to two adjacent words and cannot be treated as a phrase. As can be seen from Table 1, the proportion of non-word islands in each network can be almost ignored.

In sum, the above results have preliminarily shown that the two-character chunks with stronger internal than external tightness due to their repeated use are generally CTCWs.

**Fig 3:**
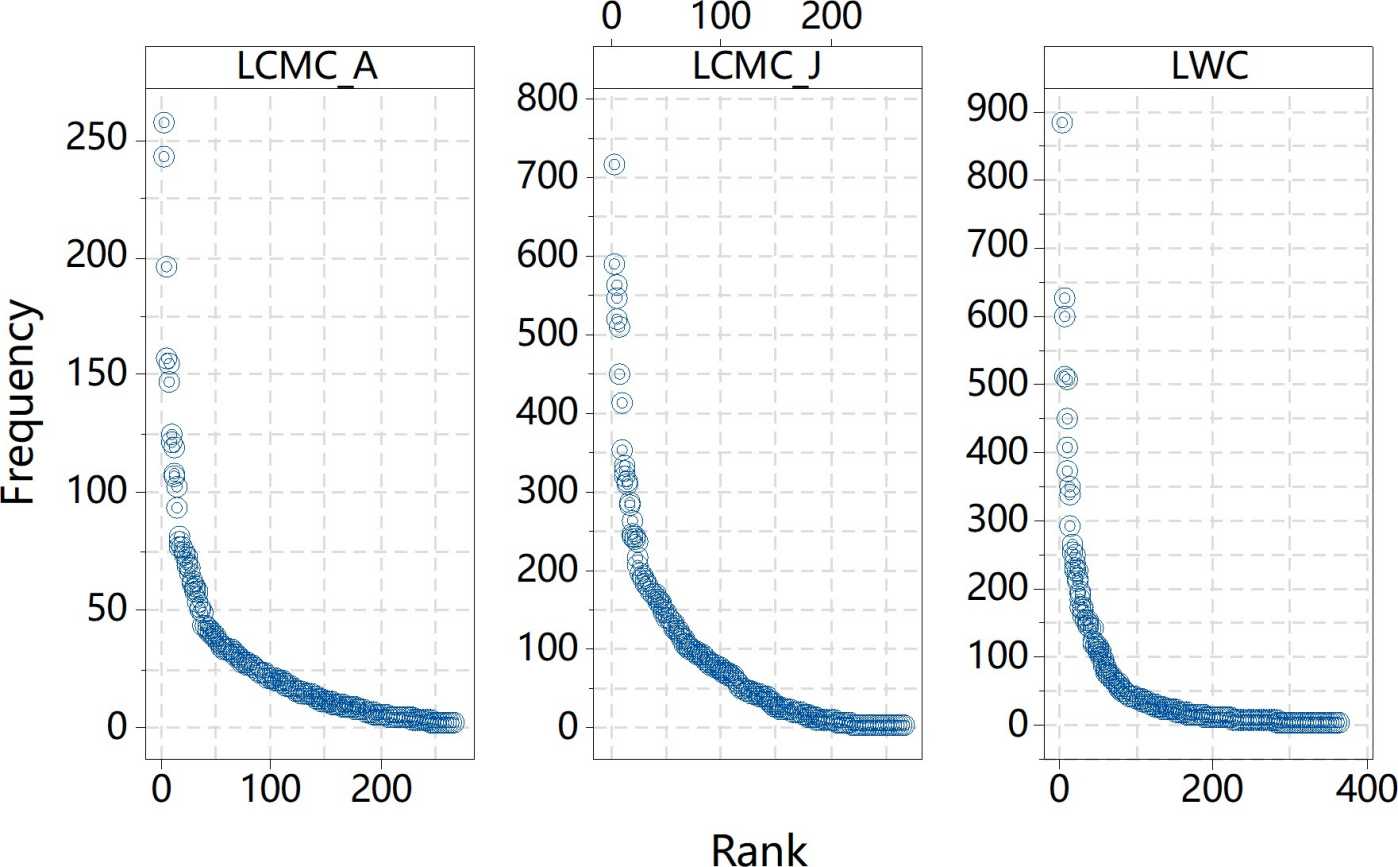
Rank-frequency distributions of CTCWs forming TNIs in the three networks.

In network analysis, the minimal weight value of the links between the component nodes of an island is termed as the *height* of the island [35]. In this study, the height of a TNI is the frequency of the corresponding chunk. In Fig 1, for instance, 语言 (yǔyán, ‘language’) as a TNI exhibits a height of 3. The distribution of word frequency in natural language can be captured by power law [33], which is known as Zipf’s Law. In other words, when the words in a language are ranked in descending order of frequency, word frequency (*f*) and rank (*r*) generally follows a power-law relationship *f ~ r*^*-γ*^. The rank-frequency distribution obtained is extremely uneven: a rather limited number of words having extremely high levels of frequency while the majority of words having rather low levels of frequency. Most CTCWs have very low levels of frequency, which however does not deny their status as words. Fig 3 displays the rank-frequency distributions of Type-1 TNIs, i.e., the CTCWs, in the three networks (see Appendices A-C in S1 File). All the plots in Fig 3 exhibit Zipf-like distributions, with word frequency dropping abruptly to a low level and then decreasing rather slowly as word rank increases. It might be unnecessary to test statistically how well the distributions fit power law. The most important message from the distributions, however, is that the TNIs extracted covered CTCWs with vastly different frequency levels. In other words, there is no necessary connection between the formation of a TNI and the frequency level of the corresponding CTCW.

The definition of islands constitutes a rather rigorous criterion for detection of two-character chunks with stronger internal than external tightness, for an island is a local sub-network with the greatest link weight values. The same character (e.g., 语 (yǔ)) may occur in more than one word (e.g., 语言 (yǔyán, ‘language’) and语法 (yǔfǎ, ‘grammar’)). However, a TNI *uv* in a given network will prevent the other character bigrams containing *u* or *v* in the same network from forming islands, even though some of them might also be CTCWs. In addition, a CTCW which occurs in particular immediate contexts (e.g., one that happens to occur always between the same neighboring characters in a particular text) may also fail to form a TNI. It follows that island extraction as conducted in this study will inevitably miss a number of CTCWs in a network.

The CTCWs that are missed by island extraction are worthy of investigation, especially whether they can form TNIs with the change of their immediate contexts. Actual language use is rich and flexible. The same is true of the immediate context of a CTCW. A body of authentic language use, regardless of its size, only constitutes a fragment of language experience. Given a particular fragment of language use (e.g., the sub-corpora LCMC_A and LCMC_J), a CTCW may fail to form a TNI in its immediate context. However, other fragments of language use may provide appropriate immediate contexts where the CTCW may well form a TNI. This is due to the nature of CTCWs as reusable character bigrams. This reusability gives rise to a frequency effect, which makes a CTCW a structural whole with stronger internal than external tightness in appropriate immediate contexts. In order to illustrate this point, the TNIs extracted from three sub-networks of Network LCMC_J were examined. These three sub-networks were network models constructed respectively on the basis of three text samples selected at random from sub-corpus LCMC_J (with 583, 1,495, and 3,353 character tokens, respectively). The three sub-networks are labeled as Networks LCMC_J_1, LCMC_J_2, and LCMC_J_3 according to their sizes (with Network LCMC_J_1 being the smallest).

Table 2 displays statistics of the TNIs in the three sub-networks (see Appendix D in S1 File). The TNIs are generally CTCWs in the three networks, especially Networks LCMC_J_2 and LCMC_J_3. The relatively low proportion of CTCWs in Network LCMC_J_1 is probably due to the small size of the text sample on the basis of which it was constructed. In each of the three sub-networks, there were CTCWs which failed to form TNIs in Network LCMC_J (see Table 2 for their numbers). For instance, 数量 (shùliàng, ‘quantity’) failed to form a TNI in Network LCMC_J due to the formation of 数据 (shùjù, ‘data’) as a TNI. However, 数量 (shùliàng, ‘quantity’) formed a TNI due to the change of its immediate context in Network LCMC_J_1. If text samples of various sizes can be selected repeatedly from a large Chinese corpus, then the TNIs extracted from these samples will cover the overwhelming majority, if not all, of the CTCWs (with a frequency of at least 2) in the corpus. Therefore, even with the rather rigorous criterion for island extraction, a CTCW still has chances to form a TNI thanks to its repeated use in appropriate immediate contexts.

**Table 2:** Basic information of TNIs extracted from three sub-networks of Network LCMC_J.

Network	CTCWs	Word-like chunks	Non-word chunks	CTCWs not extracted from Network LCMC_J	Total
LCMC_J_1	17 (89.47%)	2 (10.53%)	0	10	19
LCMC_J_2	37 (92.50%)	3 (7.50%)	0	28	40
LCMC_J_3	65 (94.20%)	4 (5.80%)	0	41	69

To recap, CTCWs have been examined, in their immediate contexts, through network analysis based on the notion of islands. There are three major findings: (1) the TNIs in the linguistic networks based on Chinese characters and their co-occurrence relations are generally CTCWs; (2) a CTCW of any frequency level (usually at least 2) may form a TNI; and (3) any CTCW (usually with a frequency of at least 2) has the potential to form a TNI in appropriate immediate contexts.

## 6. Discussion

In a wide range of research fields, the notion of islands has helped researchers to identify, in systems of various types, cohesive groups which are meaningful in one way or another [40–42]. In this study, linguistic networks as models of actual language use of Chinese have been constructed and analyzed, with Chinese characters as nodes, their co-occurrence relations as links, and the co-occurrence frequencies as link weights. It has been found that the TNIs extracted from these networks are generally CTCWs. Furthermore, it has been shown that a CTCW always has the potential to form a TNI in appropriate immediate contexts. The findings of this study, therefore, suggest a highlighting mechanism of CTCWs in their immediate contexts. While this highlighting mechanism is by itself a statistical (frequency-based) effect, it may have a bearing on the emergence of CTCWs, especially when viewed from the perspective of attention/activation.

An island as a cohesive whole is ‘a local summit in the network’, which is ‘raised above its immediate surroundings’ [35: 129]. A CTCW, by forming an island in appropriate immediate contexts, can highlight itself as a structural whole with stronger internal tightness (as can be seen from the case of 语言 (yǔyán, ‘language’) in Fig 1). In this way, it can form a local peak in terms of attention/activation level in language experience and can be readily perceived and represented as a whole. The ever-changing active areas of language experience [6: 74] give rise to great flexibility of immediate contexts of the CTCWs, so that any CTCW can have the opportunities to form a local peak of attention/activation level in language experience. The repeated highlighting of a CTCW in appropriate immediate contexts can entrench a CTCW as a local peak in language experience, so that it can it can establish and maintain its status as a structural whole. Similarly, this highlighting mechanism may also contribute to the holistic representation of a two-character phrase (e.g., those extracted from the network models). Considering that two-character phrases constitute an important source of new CTCWs in Chinese [43], this mechanism may also play a role in the future lexicalization of some of these phrases.

Moreover, the highlighting mechanism points to a contextual/networks approach to co-occurrence frequency. The findings indicate that for a CTCW to be highlighted as a cohesive whole in its immediate context, its component characters’ co-occurrence frequency does not need to reach a particular threshold. Such a non-threshold effect of frequency is also found in other studies [44–46]. What matters most to the highlighting of a CTCW has been found to be the advantage of its component characters’ co-occurrence frequency in the immediate context. Such an advantage suggests that a CTCW can be used repeatedly in diversified contexts, highlighting it as cohesive whole in its immediate surroundings. In other words, co-occurrence frequency studied in isolation is insufficient in accounting for the chunking of Chinese characters into CTCWs; rather, it needs to be examined in its immediate contexts. Such importance of contextual information has been appreciated by a growing body of usage-based research. For instance, empirical inquiries [47, 48] have shown that contextual diversity facilitates the processing and acquisition of lexical and sub-lexical units better than their frequencies alone.

This contextual/networks approach may also apply to other structural patterns (based on the chunking of lower-level units), with other quantitative measures of co-occurrence strength possibly involved. The above discussion is generally speculative. In future research, it is necessary to investigate the cognitive reality of the highlighting mechanism. For instance, will the two-character sequences that form TNIs differ in the shape of P300 from those that do not, considering that P300 is related to attention and activation of working memory [49, 50]?

## 7. Conclusions

With appropriate models and analytical techniques of linguistic networks, this study focuses on the emergence of CTCWs (as structural units based on the chunking of their component characters) in the actual use of modern Chinese, which manifests itself as continuous streams of Chinese characters. Based on a relational view of co-occurrence frequency, two-character chunks with their component characters co-occurring more frequently than they do with the other characters (i.e., two-node islands in terms of network analysis) were extracted from linguistic network models of authentic language use of modern Chinese, and the relationship between the TNIs extracted and CTCWs was determined. It has been shown that the TNIs extracted are generally CTCWs and a CTCW, regardless of its frequency level (usually above 2), always has the potential to form a TNI in appropriate immediate contexts.

The findings of this study have shed some light on the emergent mechanism of CTCWs in language use. This mechanism helps to highlight a CTCW as a structural whole in its immediate context, and thus may play a vital role in the formation and acquisition of CTCWs in actual language use. This mechanism may also throw some light on the emergence of other structural patterns (with the chunking of specific linguistic units as their basis). Moreover, the findings may help to further understand the role of frequency (and probably other types of co-occurrence strength) in linguistic emergence, especially the specific scope of the role.

Methodologically speaking, this study has further shown the value of linguistic networks in usage-based research into the structural patterns of language. To be specific, linguistic network models based on authentic language data may provide an effective means to researching the emergence of structural patterns of human language. The findings of this study have also confirmed the advantages of linguistic networks in handling relational linguistic phenomena in authentic language use.

This study is still preliminary and further researches are wanted. For instance, the highlighting mechanism is still at the computational level [51] in that it is based on statistics of actual language use and its cognitive realism needs to be determined by researches concerning the psychological and neural levels. In addition, considering the advantages of linguistic networks in dealing with relational linguistic phenomena, network-based investigations can be conducted to unveil the formation of other structural patterns of human language.

## Supporting information

S1 File (DOCX)
