# Supplementary material for: Linguistic emergence from a networks approach: The case of modern Chinese two-character words
Source: PLoS One. 2021 Nov 11;16(11):e0259818. doi: 10.1371/journal.pone.0259818 (PMC8584675; doi:10.1371/journal.pone.0259818)
Supplement: S1 File — (DOCX) [file pone.0259818.s001.docx]

**S1 File**

**Appendix A: Two-Node islands (TNIs) extracted from Network LCMC_A**

Table A1. Two-character words (269 in total, with proper nouns asterisked)

| **TNI** | **Phonetic transcription** | **English gloss** | **Height** |
| --- | --- | --- | --- |
| 我们 | wǒmen | we; us | 257 |
| 一个 | yīgè | a; an; one | 243 |
| 记者 | jìzhě | journalist | 196 |
| 企业 | qǐyè | enterprise; business | 196 |
| 经济 | jīngjì | economic; economy | 157 |
| 生产 | shēngchǎn | production; produce; manufacture | 155 |
| 公司 | gōngsī | company; corporation; firm | 147 |
| 市场 | shìchǎng | market; marketplace | 125 |
| 国家 | guójiā | country; state; nation | 121 |
| 同志 | tóngzhì | comrade | 119 |
| 技术 | jìshù | technology; skill | 108 |
| 没有 | méiyǒu | not have; be without | 107 |
| 工作 | gōngzuò | work; job | 102 |
| 发展 | fāzhǎn | develop; growth | 94 |
| 世界 | shìjiè | world; global; earth | 81 |
| 领导 | lǐngdǎo | lead; leader | 79 |
| 干部 | gànbù | cadre | 77 |
| 政府 | zhèngfǔ | government | 77 |
| 进行 | jìnxíng | in progress; carry out | 77 |
| 什么 | shénme | what | 75 |
| 北京 | běijīng | capital city of China | 74 |
| 就是 | jiùshì | exactly; namely | 73 |
| 现在 | xiànzài | now; at present; today | 70 |
| 问题 | wèntí | question; problem | 69 |
| 自己 | zìjǐ | oneself; self | 68 |
| 去年 | qùnián | last year | 66 |
| 上海 | shànghǎi | a city of China | 62 |
| 研究 | yánjiū | study; research | 61 |
| 资金 | zījīn | fund; capital | 60 |
| 可以 | kěyǐ | can; may | 59 |
| 开始 | kāishǐ | begin; start | 58 |
| 大学 | dàxué | university; college | 58 |
| 建设 | jiànshè | build; construct | 54 |
| 社会 | shèhuì | society; community | 51 |
| 时间 | shíjiān | time | 50 |
| 汽车 | qìchē | automobile; motor vehicle; car | 50 |
| *小平 | xiǎopíng | someone’s name | 49 |
| 战士 | zhànshì | warrior; soldier | 44 |
| *深圳 | shēnzhèn | place name | 44 |
| 销售 | xiāoshòu | sales; sell | 43 |
| 竞争 | jìngzhēng | compete; contend | 42 |
| 精神 | jīngshén | spirit; mind | 42 |
| 库存 | kùcún | inventory; stock; repertory | 41 |
| 电子 | diànzǐ | electron, electronic | 41 |
| 告诉 | gàosù | tell | 40 |
| 增长 | zēngzhǎng | increase; grow; rise | 40 |
| 辽宁 | liáoníng | a province of China | 39 |
| 群众 | qúnzhòng | the masses; common people | 39 |
| 下午 | xiàwǔ | afternoon | 39 |
| 方面 | fāngmiàn | aspect; side | 38 |
| 总统 | zǒngtǒng | president | 37 |
| 负责 | fùzé | be responsible for; be in charge of | 37 |
| 情况 | qíngkuàng | situation; circumstances | 36 |
| 主要 | zhǔyào | main; major | 36 |
| *李放 | lǐfàng | someone’s name | 35 |
| 百分 | bǎifēn | percent | 35 |
| 效益 | xiàoyì | benefit; effectiveness | 34 |
| 计划 | jìhuà | plan; project | 34 |
| 许多 | xǔduō | many; much | 34 |
| 服务 | fúwù | service; serve | 34 |
| 参加 | cānjiā | attend; join; participate in | 33 |
| 组织 | zǔzhī | organization; organize; tissue | 33 |
| 革命 | gémìng | revolution; revolutionary | 33 |
| 苏联 | sūlián | Soviet Union | 33 |
| 沈阳 | shěnyáng | a city of China | 32 |
| 目前 | mùqián | currently; at present | 32 |
| 管理 | guǎnlǐ | manage; supervise | 31 |
| 日本 | rìběn | Japan | 31 |
| 历史 | lìshǐ | history | 30 |
| 而且 | érqiě | and; furthermore | 30 |
| 由于 | yóuyú | thanks to; as a result of; due to | 30 |
| 调查 | diàochá | investigate; examine | 29 |
| 医院 | yīyuàn | hospital | 29 |
| 质量 | zhìliàng | quality; mass | 28 |
| 积压 | jīyā | overstock | 28 |
| 交易 | jiāoyì | transaction; trade; deal | 28 |
| 准备 | zhǔnbèi | prepare; get ready | 28 |
| 结构 | jiégòu | structure; composition; construction | 28 |
| 激动 | jīdòng | excited; excitement | 27 |
| 价格 | jiàgé | price | 27 |
| 高兴 | gāoxìng | happy; glad | 27 |
| 山东 | shāndōng | a province of China | 27 |
| 宣布 | xuānbù | declare; proclaim | 27 |
| 铁路 | tiělù | railway; railroad; rail | 26 |
| 信息 | xìnxī | information; message; news | 26 |
| 基础 | jīchǔ | foundation; basis; base | 26 |
| 坚持 | jiānchí | insist on; stick to; adhere to | 25 |
| 纷纷 | fēnfēn | one after another; in succession | 25 |
| 垃圾 | lājī | garbage; rubbish; trash | 24 |
| 军事 | jūn shì | military affairs | 23 |
| 旅客 | lǚkè | hotel guest; traveller; passenger | 23 |
| 回答 | huídá | answer; reply; response | 23 |
| *延安 | yán’ān | a city of China | 23 |
| 收入 | shōurù | income; revenue | 23 |
| 变化 | biànhuà | change; variety; vary | 23 |
| 欧洲 | ōuzhōu | Europe | 22 |
| *铜陵 | tónglíng | a city of China | 22 |
| 冰箱 | bīngxiāng | refrigerator; fridge | 21 |
| 承包 | chéngbāo | contract (with; for) | 21 |
| 介绍 | jièshào | introduce; present; recommend | 21 |
| 欢迎 | huānyíng | welcome; greet | 21 |
| 必须 | bìxū | must; necessary; have to | 21 |
| 消费 | xiāofèi | consumption; consume | 21 |
| 保证 | bǎozhèng | guarantee; ensure; assure | 20 |
| 武汉 | wǔhàn | a city of China | 20 |
| 母亲 | mǔqīn | mother | 20 |
| *鞍钢 | ān’gāng | a corporation’s name | 20 |
| 困难 | kùnnán | difficulty; hard; trouble | 20 |
| 连续 | liánxù | continuing; running; continuous; successive | 20 |
| 柜台 | guìtái | counter; bar | 20 |
| 稳定 | wěndìng | stable; steady; stabilization | 20 |
| 优秀 | yōuxiù | excellent; outstanding; excellence | 19 |
| *兰考 | lánkǎo | a city of China | 19 |
| 香港 | xiānggǎng | Hong Kong | 18 |
| 贷款 | dàikuǎn | loan; credit; provide a loan | 18 |
| 棉花 | miánhuā | cotton | 18 |
| 训练 | xùnliàn | train; practice; drill | 18 |
| 掌声 | zhǎngshēng | applause; clapping | 18 |
| 体育 | tǐyù | sports; physical culture; physical training | 18 |
| 控制 | kòngzhì | control; dominate; regulate | 17 |
| 希望 | xīwàng | hope; want; wish | 17 |
| 应用 | yìngyòng | application; use; apply | 16 |
| 材料 | cáiliào | material; data | 16 |
| 秩序 | zhìxù | order; system; sequence | 16 |
| 妇女 | fùnǚ | woman; feme; femineity | 16 |
| 原因 | yuányīn | reason; cause | 16 |
| 采访 | cǎifǎng | have an interview with; make inquiries | 16 |
| 批评 | pīpíng | criticism; criticize; comment | 15 |
| 刚刚 | gānggāng | just; only; just now | 15 |
| 春节 | chūnjiē | Chinese New Year; Spring Festival | 15 |
| 妈妈 | māma | mom; mum | 15 |
| 比赛 | bǐsài | match; competition; contest | 15 |
| 矛盾 | máodùn | contradiction; contradict | 15 |
| *雷锋 | léifēng | someone’s name | 14 |
| 左右 | zuǒyòu | about; around; control | 14 |
| 四川 | sìchuān | a province of China | 14 |
| 帮助 | bāngzhù | help; assistance; assist | 14 |
| 根据 | gēnjù | on the basis of; according to; in the light of | 14 |
| 临床 | línchuáng | clinical | 13 |
| 麻风 | máfēng | leprosy | 13 |
| 艰苦 | jiānkǔ | arduous; difficult; hard | 13 |
| 贡献 | gòngxiàn | contribution; contribute; dedicate | 13 |
| 街头 | jiētóu | street; street corner | 12 |
| 申请 | shēnqǐng | application; apply; apply for | 12 |
| 冷轧 | lěngzhá | cold rolling | 12 |
| 薄板 | báobǎn | thin plate | 12 |
| 损失 | sǔnshī | loss; lose | 11 |
| 攻击 | gōngjī | attack; assault | 11 |
| 星级 | xīngjí | star-rated | 11 |
| 紧急 | jǐnjí | urgent; emergent; critical | 11 |
| 咨询 | zīxún | consult; seek advice from | 11 |
| 购买 | gòumǎi | buy; purchase | 11 |
| 等待 | děngdài | wait; await | 11 |
| 周围 | zhōuwéi | around; round; about | 11 |
| 选择 | xuǎnzé | choice; choose; select | 11 |
| 洗衣 | xǐyī | wash clothes | 11 |
| 丰富 | fēngfù | rich; abundant; enrich | 10 |
| 犯罪 | fànzuì | commit a crime | 10 |
| 签订 | qiāndìng | conclude and sign | 10 |
| 维修 | wéixiū | keep in (good) repair | 10 |
| 爸爸 | bàba | papa; dad | 10 |
| 沙丘 | shāqiū | sand dune | 10 |
| 防汛 | fángxùn | flood prevention (control) | 10 |
| 恢复 | huīfù | recovery; recover; renew | 10 |
| 友谊 | yǒuyì | friendship | 10 |
| 茶叶 | cháyè | tea-leaves | 9 |
| 拍卖 | pāimài | auction; sell something at auction | 9 |
| 葡萄 | pútáo | grape | 9 |
| 煤炭 | méitàn | coal | 9 |
| 预测 | yùcè | forecast; calculate | 9 |
| 英雄 | yīngxióng | hero | 9 |
| 歹徒 | dǎitú | scoundrel; ruffian | 9 |
| 悄悄 | qiāoqiāo | quietly; on the quiet | 9 |
| 勃勃 | bóbó | thriving; vigorous | 9 |
| 转移 | zhuǎnyí | transfer; shift; change | 9 |
| 储蓄 | chǔxù | savings; deposit; save | 9 |
| 速度 | sùdù | speed; velocity | 9 |
| *刘帅 | liúshuài | someone’s name | 8 |
| 仔细 | zǐxì | careful; attentive | 8 |
| 抵押 | dǐyā | mortgage; pledge | 8 |
| 膨胀 | péngzhàng | swell; expand | 8 |
| 培养 | péiyǎng | train; develop; foster | 8 |
| 健康 | jiànkāng | health; healthy; wholesome | 8 |
| 呼唤 | hūhuàn | call; shout to | 8 |
| 啤酒 | píjiǔ | beer | 8 |
| 屏幕 | píngmù | screen | 8 |
| 永远 | yǒngyuǎn | forever; always | 7 |
| 疲软 | píruǎn | fatigued and weak | 7 |
| 辉煌 | huīhuáng | glorious; splendid; magnificent | 7 |
| 清楚 | qīngchǔ | clear; distinct; lucid | 7 |
| 录音 | lùyīn | tape; record; sound recording | 7 |
| 配套 | pèitào | mating; form a complete set | 7 |
| 婆婆 | pópo | husband’s mother | 7 |
| 爆破 | bàopò | blast; burst; blow up | 7 |
| 废墟 | fèixū | ruins; wasteland | 6 |
| 档案 | dàng’àn | files; archives | 6 |
| 喇叭 | lǎbā | horn; trumpet; loudspeaker | 6 |
| 玻璃 | bōlí | glass | 6 |
| *鹰潭 | yīngtán | a city of China | 6 |
| 默默 | mòmò | silently; quietly | 6 |
| 涌潮 | yǒngcháo | sea bore | 6 |
| 逐渐 | zhújiàn | gradually; little by little | 6 |
| 倒爷 | dǎoyé | wheeler and dealer | 6 |
| 缓缓 | huǎnhuǎn | slowly; gradually | 6 |
| 拼搏 | pīnbó | go all out in work; combat (with; against) | 6 |
| 截瘫 | jiétān | paraplegia | 6 |
| 鼓励 | gǔlì | encourage; incite | 6 |
| 蜡烛 | làzhú | candle | 5 |
| 卧铺 | wòpù | sleeper; couchette | 5 |
| 扬扬 | yángyáng | triumphantly; complacently | 5 |
| 驾驶 | jiàshǐ | drive; pilot; steer | 5 |
| 暴雨 | bàoyǔ | rainstorm | 5 |
| 短短 | duǎnduǎn | short; little | 5 |
| 滔滔 | tāotāo | surging; torrential; billowy | 5 |
| 避免 | bìmiǎn | avoid; prevent | 5 |
| 途径 | tújìng | way; road; avenue | 5 |
| 忠诚 | zhōngchéng | loyal; faithful; fidelity | 5 |
| 翻砂 | fānshā | molding; casting | 5 |
| 奔驰 | bēnchí | run quickly; gallop | 5 |
| 熟悉 | shúxī | be familiar with; have an intimate knowledge of | 5 |
| 泡桐 | pāotóng | paulownia | 5 |
| 匆匆 | cōngcōng | hurriedly; in a rush | 5 |
| 岂止 | qǐzhǐ | not only/merely; more than | 4 |
| 茫茫 | mángmáng | vast; boundless | 4 |
| 锻炼 | duànliàn | take exercise; have physical training | 4 |
| 锅炉 | guōlú | boiler; furnace | 4 |
| 仿佛 | fǎngfú | seem; as if | 4 |
| 恐怕 | kǒngpà | perhaps; fear; I’m afraid | 4 |
| 慢慢 | mànmàn | slowly; gradually | 4 |
| 伪劣 | wěiliè | false and inferior | 4 |
| 镶嵌 | xiāngqiàn | inset; mosaic | 3 |
| 纵横 | zònghéng | freely; vertically and horizontally | 3 |
| 挖掘 | wājué | dig; tap; excavate | 3 |
| 抽屉 | chōutì | drawer | 3 |
| 钥匙 | yàoshi | key | 3 |
| 尖锐 | jiānruì | sharp-pointed; keen | 3 |
| 智慧 | zhìhuì | wisdom; intelligence | 3 |
| 呜咽 | wūyè | sob; whimper | 3 |
| 草丛 | cǎocóng | brushwood; thick growth of grass | 3 |
| 鞠躬 | jūgōng | bow; bow down | 3 |
| 刺槐 | cìhuái | locust (tree) | 3 |
| 狭窄 | xiázhǎi | narrow; cramped | 3 |
| 诞辰 | dànchén | birthday | 3 |
| 哀悼 | āidào | lament; grieve over | 3 |
| 刷刷 | shuāshuā | an onomatopoeic word | 3 |
| 喷浆 | pēnjiāng | guniting; whitewashing | 3 |
| 贪污 | tānwū | corruption; embezzlement; embezzle | 2 |
| 枢纽 | shūniǔ | hub; hinge; pivot | 2 |
| 辣椒 | làjiāo | chilli; hot pepper | 2 |
| 喀嚓 | kāchā | crack; snap | 2 |
| 逃窜 | táocuàn | run away; flee in disorder | 2 |
| 伦敦 | lúndūn | London | 2 |
| 呵呵 | hēhē | an onomatopoeic word for laughter | 2 |
| 肿瘤 | zhǒngliú | tumor | 2 |
| 荫庇 | yīnbì | protection by one’s elders or ancestors | 2 |
| 萦绕 | yíngrào | linger; hover | 2 |
| 甲苯 | jiǎběn | methylbenzene; toluene | 2 |
| 叮嘱 | dīngzhǔ | exhort; repeatedly advise | 2 |
| 蛋糕 | dàngāo | cake | 2 |
| 硫酸 | liúsuān | sulphuric acid | 2 |
| 嘻嘻 | xīxī | an onomatopoeic word for laughter | 2 |
| 毒蛇 | dúshé | serpent; poisonous (venomous) snake | 2 |
| 瘢痕 | bānhén | scar; cicatrix | 2 |
| 寥寥 | liáoliáo | few; scanty | 2 |
| 疙瘩 | gēda | lump; pimple; knot | 2 |
| 捐赠 | juānzèng | donation; donate | 2 |
| 祈祷 | qídǎo | pray; say one’s prayers | 2 |
| 徘徊 | páihuái | hover; linger about | 2 |
| 挨饿 | áiè | suffer from hunger; starve | 2 |

Table A2. Word-like chunks (6 in total)

| **TNI** | **Phonetic transcription** | **English gloss** | **Height** |
| --- | --- | --- | --- |
| 万元 | wànyuán | ten thousand Yuan RMB | 70 |
| 坟岗 | fén’gǎng | grave mound | 10 |
| 无轨 | wúguǐ | without rail | 8 |
| 玉璧 | yùbì | round flat piece of jade | 6 |
| 瑶胞 | yáobāo | the Yao people (a Chinese ethnic group) | 4 |
| 盐硷 | yánjiǎn | saline-alkali | 4 |

Table A3. Non-word chunks (1 in total)

| **TNI** | **Phonetic transcription** | **Height** |
| --- | --- | --- |
| 地说 | deshuō | 42 |

**Appendix B: Two-Node islands (TNIs) extracted from Network LCMC_J**

Table B1. Two-character words (267 in total, with proper nouns asterisked)

| **TNI** | **Phonetic transcription** | **English gloss** | **Height** |
| --- | --- | --- | --- |
| 系统 | xìtǒng | system; systematic | 715 |
| 经济 | jīngjì | economic; economy | 589 |
| 社会 | shèhuì | society; community | 563 |
| 发展 | fāzhǎn | develop; growth | 545 |
| 科学 | kēxué | science; scientific knowledge | 518 |
| 一个 | yīgè | a; an; one | 508 |
| 技术 | jìshù | technology; skill | 450 |
| 问题 | wèntí | question; problem | 411 |
| 研究 | yánjiū | study; research | 351 |
| 生产 | shēngchǎn | production; produce; manufacture | 333 |
| 语言 | yǔyán | language | 325 |
| 结构 | jiégòu | structure; composition; construction | 319 |
| 不同 | bùtóng | difference; distinct | 311 |
| 我们 | wǒmén | we; us | 310 |
| 可以 | kěyǐ | can; may | 287 |
| 进行 | jìnxíng | in progress; carry out | 282 |
| 工程 | gōngchéng | engineering; project | 264 |
| 理论 | lǐlùn | theory; exoterica | 246 |
| 自然 | zìrán | nature; naturally; natural world | 242 |
| 方面 | fāngmiàn | aspect; side | 242 |
| 作用 | zuòyòng | effect; function | 239 |
| 价值 | jiàzhí | worth; value | 236 |
| 主要 | zhǔyào | main; major | 215 |
| 活动 | huódòng | activity; campaign; exercise | 207 |
| 影响 | yǐngxiǎng | influence; impact; affect | 195 |
| 企业 | qǐyè | enterprise; business | 195 |
| 教育 | jiàoyù | education; educate; teach | 192 |
| 世界 | shìjiè | world; global; earth | 189 |
| 标准 | biāozhǔn | standard; criterion | 186 |
| 具有 | jùyǒu | have; possess | 185 |
| 计算 | jìsuàn | calculate; compute; count | 182 |
| 观察 | guānchá | observation; observe; watch | 180 |
| 基本 | jīběn | basic; fundamental; essential; main | 178 |
| 存在 | cúnzài | exist; existence | 172 |
| 历史 | lìshǐ | history; past records | 171 |
| 由于 | yóuyú | thanks to; as a result of; due to | 170 |
| 资源 | zīyuán | resource | 169 |
| 市场 | shìchǎng | market; marketplace | 168 |
| 必须 | bìxū | must; necessary; have to | 168 |
| 因此 | yīncǐ | therefore; thus; accordingly | 165 |
| 平衡 | pínghéng | balance; equilibrium | 162 |
| 实际 | shíjì | actual; reality; fact | 158 |
| 认识 | rènshí | know; be familiar with; be acquainted with | 158 |
| 新闻 | xīnwén | news; press | 158 |
| 数据 | shùjù | data; record | 155 |
| 组织 | zǔzhī | organization; organize; tissue | 150 |
| 旅游 | lǚyóu | tourism; tour | 147 |
| 汉字 | hànzì | Chinese character | 146 |
| 之间 | zhījiān | between | 145 |
| 条件 | tiáojiàn | condition; term; requirement | 140 |
| 形成 | xíngchéng | formation; form; take shape | 139 |
| 变化 | biànhuà | change; variety; vary | 138 |
| 出现 | chūxiàn | appear; appearance; arise | 137 |
| 信息 | xìnxī | information; message; news | 135 |
| 公司 | gōngsī | company; corporation; firm | 133 |
| 对象 | duìxiàng | object; target; boyfriend; girl friend | 129 |
| 部分 | bùfēn | part; section; portion | 127 |
| 如果 | rúguǒ | if; in case; in the event of | 124 |
| 思想 | sīxiǎng | thought; idea; thinking | 124 |
| 情况 | qíngkuàng | situation; circumstances | 121 |
| 提高 | tígāo | increase; raise; enhance | 118 |
| 包括 | bāokuò | include; consist of; comprise | 116 |
| 概念 | gàiniàn | concept; conception; notion | 114 |
| 相互 | xiānghù | mutual; reciprocal; each other | 113 |
| 地区 | dìqū | area; region; district | 111 |
| 改革 | gǎigé | reform; reformation; innovation | 107 |
| 什么 | shénme | what | 106 |
| 许多 | xǔduō | many; much | 103 |
| 阶段 | jiēduàn | stage; phase; period | 101 |
| 而且 | érqiě | and; furthermore | 100 |
| 商品 | shāngpǐn | commodity; goods; merchandise | 100 |
| 比较 | bǐjiào | compare; relatively | 99 |
| 电子 | diànzǐ | electron, electronic | 98 |
| 直接 | zhíjiē | direct; straight; immediate | 97 |
| 建立 | jiànlì | establish; build; set up | 96 |
| 内容 | nèiróng | content; substance | 95 |
| 矛盾 | máodùn | contradiction; contradict | 95 |
| 口令 | kǒulìng | password; command | 94 |
| 单位 | dānwèi | unit | 94 |
| 政治 | zhèngzhì | politics; political affairs | 93 |
| 反映 | fǎnyìng | reflect; reflex | 93 |
| 层次 | céngcì | level; gradation | 91 |
| 符号 | fúhào | symbol; sign; mark | 89 |
| 小说 | xiǎoshuō | novel; fiction | 88 |
| 贸易 | màoyì | trade; commerce; traffic | 87 |
| 民族 | mínzú | nation; nationality | 83 |
| 复杂 | fùzá | complex; complicated; complexity | 82 |
| 输入 | shūrù | entry; import; input | 81 |
| 指导 | zhǐdǎo | guidance; guide; direct | 80 |
| 规律 | guīlǜ | law; rule; regular pattern | 80 |
| 原则 | yuánzé | principle; fundamental | 80 |
| 领域 | lǐngyù | field; domain; sphere | 79 |
| 编辑 | biānjí | editor; edit; compile | 78 |
| 状态 | zhuàngtài | state; status; condition | 77 |
| 细胞 | xìbāo | cell | 77 |
| 完全 | wánquán | completely; entirely; totally | 76 |
| 选择 | xuǎnzé | choice; choose; select | 76 |
| 得到 | dédào | get; obtain; receive; gain | 76 |
| 调节 | tiáojié | adjust; regulate; monitor | 74 |
| 肥胖 | féipàng | fat; obesity | 73 |
| 随着 | suízhe | along with; in the wake of; in pace with | 70 |
| 集团 | jítuán | group; clique; circle | 70 |
| 创造 | chuàngzào | create; produce; bring about | 70 |
| 环境 | huánjìng | environment; surroundings; circumstances | 68 |
| 增加 | zēngjiā | increase; raise; add | 68 |
| 脂肪 | zhīfáng | fat; axunge | 67 |
| 甚至 | shènzhì | even; (go) so far as to | 67 |
| 减少 | jiǎnshǎo | reduce; decrease; lessen | 67 |
| 森林 | sēnlín | forest; timber | 65 |
| 材料 | cáiliào | material; data; makings | 65 |
| 保持 | bǎochí | keep; hold; retain | 61 |
| 培养 | péiyǎng | train; develop; foster | 59 |
| 首先 | shǒuxiān | firstly; first; in the first place | 58 |
| 记录 | jìlù | record; take notes; keep an account of | 56 |
| 任何 | rènhé | any; whatever; whichever | 55 |
| 精神 | jīngshén | spirit; mind; essence | 51 |
| 效益 | xiàoyì | benefit; effectiveness | 50 |
| 铁芯 | tiěxīn | iron core | 50 |
| 等等 | děngděng | and so on; and so on and so forth | 48 |
| 缺乏 | quēfá | lack; deficiency; be short of | 48 |
| 消费 | xiāofèi | consumption; consume | 47 |
| 往往 | wǎngwǎng | often; frequently; sometimes | 46 |
| 普遍 | pǔbiàn | common; universal; general | 46 |
| 下降 | xiàjiàng | decline; drop; fall | 45 |
| 满足 | mǎnzú | satisfy; satisfied; content | 45 |
| 装置 | zhuāngzhì | device; installation; install | 45 |
| 竞争 | jìngzhēng | compete; contend | 44 |
| 线圈 | xiànquān | winding; loop; coil | 44 |
| 服务 | fúwù | service; serve | 43 |
| 膨胀 | péngzhàng | swell; expand | 42 |
| 测试 | cèshì | test; testing; measurement | 42 |
| 考虑 | kǎolǜ | consider; think over; take into account | 42 |
| 转换 | zhuǎnhuàn | conversion; convert; transform | 41 |
| 按照 | ànzhào | according to; in the light of | 41 |
| 错误 | cuòwù | error; wrong; mistake | 39 |
| 监督 | jiāndū | supervise; superintend; supervisor | 39 |
| 掌握 | zhǎngwò | master; grasp; know well | 39 |
| 积极 | jījí | positive; active; energetic | 38 |
| 股东 | gǔdōng | shareholder; stockholder | 38 |
| 广泛 | guǎngfàn | widely; extensive; widespread | 38 |
| 差异 | chàyì | difference; discrepancy; divergence | 36 |
| 探索 | tànsuǒ | exploration; explore; probe | 35 |
| 介绍 | jièshào | introduce; present; recommend | 33 |
| 措施 | cuòshī | measure; step | 32 |
| 连队 | liánduì | (military) company | 32 |
| 胰岛 | yídǎo | pancreatic islets | 31 |
| 丰富 | fēngfù | rich; abundant; enrich | 30 |
| 炼油 | liànyóu | oil refining | 28 |
| 欧洲 | ōuzhōu | Europe | 27 |
| 距离 | jùlí | distance; range | 27 |
| 避免 | bìmiǎn | avoid; prevent | 26 |
| 左右 | zuǒyòu | about; around; control | 25 |
| 继续 | jìxù | continue; go on | 25 |
| 色彩 | sècǎi | color; hue | 24 |
| 健康 | jiànkāng | health; healthy; wholesome | 24 |
| 背景 | bèijǐng | background; backdrop; setting | 23 |
| 购买 | gòumǎi | buy; purchase | 23 |
| 战士 | zhànshì | warrior; soldier | 23 |
| 兴趣 | xīngqù | interest; taste | 23 |
| 缩短 | suōduǎn | shorten; cut | 23 |
| 帮助 | bāngzhù | help; assistance; assist | 22 |
| 刨刀 | bào dāo | planer tool | 21 |
| 污染 | wūrǎn | pollute; contaminate | 21 |
| 希望 | xīwàng | hope; want; wish | 20 |
| 疾病 | jíbìng | disease; illness | 20 |
| 破坏 | pòhuài | destroy; damage; ruin | 20 |
| 悲剧 | bēijù | tragedy; tragic drama | 19 |
| 趋势 | qūshì | trend; tendency; current | 19 |
| 儿童 | értóng | children; enfant | 18 |
| 回答 | huídá | answer; reply; response | 18 |
| 清楚 | qīngchǔ | clear; distinct; lucid | 18 |
| 北京 | běijīng | capital city of China | 18 |
| 蛋白 | dànbái | albumen; egg white | 18 |
| 渗透 | shèntòu | permeation; permeate | 16 |
| 贯彻 | guànchè | implement; carry out; put into effect | 16 |
| 涨落 | zhǎngluò | fluctuate; ebb and flow | 16 |
| 威胁 | wēixié | threaten; menace; imperil | 16 |
| 防止 | fángzhǐ | prevent; avoid | 15 |
| 千瓦 | qiānwǎ | kilowatt | 14 |
| 紧密 | jǐnmì | close together; inseparable; thick and fast | 14 |
| 障碍 | zhàng’ài | obstacle; hinder; obstruct | 13 |
| 混乱 | hùnluàn | chaos; confusion | 13 |
| 叠韵 | diéyùn | vowel rhyme; assonance | 12 |
| 训练 | xùnliàn | train; practice; drill | 12 |
| 翻译 | fānyì | translate; translator; interpreter; interpret | 11 |
| 轮廓 | lúnkuò | outline; contour profile | 11 |
| 颠倒 | diāndǎo | put (turn) upside down; overthrow; confused | 10 |
| 玉米 | yùmǐ | corn | 10 |
| 摆脱 | bǎituō | rid; dispense; cast off | 10 |
| 宇宙 | yǔzhòu | cosmos; universe | 10 |
| 赋予 | fùyǔ | give; endow; entrust | 10 |
| 覆盖 | fùgài | cover; covering; overlap | 9 |
| 牛顿 | niúdùn | Newton | 9 |
| 死亡 | sǐwáng | death; die | 9 |
| 蔬菜 | shūcài | vegetable | 9 |
| 牺牲 | xīshēng | sacrifice | 9 |
| 浙江 | zhèjiāng | a province of China | 9 |
| 途径 | tújìng | way; road; avenue | 9 |
| 月亮 | yuèliàng | moon | 9 |
| *蔡伦 | càilún | name of a figure in Chinese history | 8 |
| 唤醒 | huànxǐng | awaken; arouse; wake up | 8 |
| 痛苦 | tòngkǔ | pain; suffering; misery | 8 |
| 乡镇 | xiāngzhèn | villages and towns | 8 |
| 恰恰 | qiàqià | precisely; just; exactly | 7 |
| 尖锐 | jiānruì | sharp-pointed; keen | 7 |
| 毁灭 | huǐmiè | destroy; ruin; exterminate | 7 |
| 春秋 | chūnqiū | age; spring and autumn; year | 7 |
| 喜欢 | xǐhuān | like; love; enjoy | 7 |
| *板垣 | bǎnyuán | a Japanese name | 6 |
| 巴黎 | bālí | Paris | 6 |
| 丝绵 | sīmián | silk wadding | 5 |
| 痕迹 | hénjì | mark; imprint; vestige | 5 |
| *凯诺 | kǎinuò | someone’s name | 5 |
| 溃疡 | kuìyáng | ulcer | 5 |
| *仡佬 | gēlǎo | name of a Chinese ethnic group | 5 |
| 朋友 | péngyǒu | friend | 5 |
| 漏掉 | lòudiào | leave out | 5 |
| 灌溉 | guàngài | irrigation; irrigate; watering | 4 |
| 妈妈 | māma | mom; mum | 4 |
| 乌龟 | wūguī | tortoise | 4 |
| 奴隶 | núlì | slave | 4 |
| 纷纷 | fēnfēn | one after another; in succession | 4 |
| 萌芽 | méngyá | bud; sprout | 4 |
| 恐怕 | kǒngpà | perhaps; fear; I’m afraid | 4 |
| 陶冶 | táoyě | edify; mold | 4 |
| 骄傲 | jiāoào | proud; arrogant; conceited | 4 |
| 禁忌 | jìnjì | taboo; avoid; abstain from | 4 |
| 碰撞 | pèngzhuàng | impact; collide; run into | 4 |
| 藩篱 | fānlí | hedge; fence | 3 |
| 饥饿 | jī’è | hunger; starvation; famine | 3 |
| 欣赏 | xīnshǎng | appreciate; enjoy; admire | 3 |
| 姊妹 | zǐmèi | elder and younger sisters | 3 |
| 卵巢 | luǎncháo | oarium | 3 |
| 钥匙 | yàoshi | key | 3 |
| 螃蟹 | pángxiè | crab | 3 |
| 隧洞 | suìdòng | tunnel | 3 |
| 隐蔽 | yǐnbì | concealment; conceal; hide | 3 |
| 胸襟 | xiōngjīn | mind; breadth of mind | 3 |
| 孔雀 | kǒngquè | peacock | 3 |
| 谨慎 | jǐnshèn | cautious; careful; prudent | 3 |
| 喇嘛 | lǎma | Lama | 3 |
| 橱窗 | chúchuāng | showcase | 3 |
| 堆栈 | duīzhàn | stack | 3 |
| 淘汰 | táotài | eliminate; be sifted out | 3 |
| 奥秘 | àomì | secret | 3 |
| 驾驭 | jiàyù | control; master; rein | 3 |
| 婆婆 | pópo | husband’s mother | 3 |
| 肺泡 | fèipào | alveolus pulmonis | 3 |
| 酝酿 | yùnniàng | brew; ferment | 3 |
| 屏幕 | píngmù | screen | 3 |
| 震撼 | zhènhàn | shock; shake; rock | 2 |
| 鹦鹉 | yīngwǔ | parrot | 2 |
| 捕捉 | bǔzhuō | catch; seize | 2 |
| 闪烁 | shǎnshuò | flicker; twinkle | 2 |
| *仓颉 | cāngjié | name of a figure in Chinese history | 2 |
| 桎梏 | zhìgù | fetters and handcuffs; shackles | 2 |
| 睾丸 | gāowán | testicle | 2 |
| 圆弧 | yuánhú | arc | 2 |
| 猖狂 | chāngkuáng | furious; savage | 2 |
| 浴缸 | yùgāng | bathtub | 2 |
| 愚蠢 | yúchǔn | stupid; foolish; silly | 2 |
| *伏羲 | fúxī | name of a figure in Chinese history | 2 |
| 潇洒 | xiāosǎ | natural and unrestrained | 2 |
| 吉凶 | jíxiōng | good or ill luck | 2 |
| 豺狼 | cháiláng | jackals and wolves; cruel and evil people | 2 |
| 褒贬 | bāobiǎn | pass judgment on | 2 |
| 朦胧 | ménglóng | dim; obscure | 2 |

Table B2. Word-like chunks (8 in total)

| **TNI** | **Phonetic transcription** | **English gloss** | **Height** |
| --- | --- | --- | --- |
| 这种 | zhèzhǒng | this kind | 253 |
| 马克 | mǎkè | a part of word, as in 马克思 (mǎkèsī, Marx) and 拉马克 (lāmǎkè, Lamarch) | 30 |
| 麦胚 | màipēi | wheat germ | 27 |
| 铃虫 | língchóng | a part of word, as in 棉铃虫 (miánlíngchóng, bollworm) and 红铃虫 (hónglíngchóng, red ballworm) | 26 |
| 斯坦 | sītǎn | a part of word, as in 爱因斯坦 (àiyīnsītǎn, Einstein) and 维特根斯坦 (wéitègēnsītǎn, Wittgenstein) | 13 |
| 沃尔 | wòěr | a part of word, as in 沃尔夫 (wòěrfū, Wolff) and 沃尔什 (wòěrshí, Walsh) | 7 |
| 阿拉 | ālā | a part of word, as in 阿拉伯 (ālābó, Arab) and 阿拉坦 (ālātǎn, Alatan) | 6 |
| 蓝紫 | lánzǐ | bluish violet | 3 |

Table B3. Non-word chunks (1 in total)

| **TNI** | **Phonetic transcription** | **Height** |
| --- | --- | --- |
| 中的 | zhōngde | 196 |

**Appendix C: Two-Node islands (TNIs) extracted from Network LWC**

Table C1. Two-character words (363 in total, with proper nouns asterisked)

| **TNI** | **Phonetic transcription** | **English gloss** | **Height** |
| --- | --- | --- | --- |
| *微博 | wēibó | name of a Chinese social media platform | 882 |
| 一个 | yīgè | a; an; one | 626 |
| 自己 | zìjǐ | oneself; self | 597 |
| 哈哈 | hāhā | an onomatopoeic word for laughter | 509 |
| 什么 | shénme | what | 508 |
| 分享 | fēnxiǎng | share | 449 |
| 今天 | jīntiān | today | 405 |
| 获得 | huòdé | acquire; gain; obtain | 373 |
| 没有 | méiyǒu | not have; be without | 347 |
| 不是 | bùshì | be not; fault | 339 |
| 超过 | chāoguò | exceed; outnumber | 291 |
| 可以 | kěyǐ | can; may | 266 |
| 知道 | zhīdào | know; realize; be aware of | 252 |
| 现在 | xiànzài | now; at present; today | 252 |
| 时候 | shíhou | time | 248 |
| 朋友 | péngyǒu | friend | 236 |
| 大家 | dàjiā | everybody; great master | 231 |
| 速度 | sùdù | speed; velocity | 227 |
| 开始 | kāishǐ | begin; start | 224 |
| 客户 | kèhù | customer; client | 215 |
| 喜欢 | xǐhuān | like; love; enjoy | 209 |
| 啊啊 | àà | an interjection | 196 |
| 希望 | xīwàng | hope; want; wish | 192 |
| 生活 | shēnghuó | life; live | 185 |
| 幸福 | xìngfú | happy; happiness; well-being | 172 |
| 推荐 | tuījiàn | recommend; recommendation | 172 |
| 感觉 | gǎnjué | sense perception; sensation; feeling | 169 |
| 地址 | dìzhǐ | address | 162 |
| 如果 | rúguǒ | if; in case; in the event of | 154 |
| 世界 | shìjiè | world; global; earth | 154 |
| 因为 | yīnwéi | because; for; on account of | 152 |
| 游戏 | yóuxì | game | 152 |
| 已经 | yǐjīng | already | 150 |
| 三国 | sānguó | a period in Chinese history | 146 |
| 看到 | kàndào | see; catch sight of | 146 |
| 终于 | zhōngyú | at last; in the end; finally | 142 |
| 手机 | shǒujī | mobile phone | 142 |
| 新年 | xīnnián | new year | 141 |
| 城市 | chéngshì | city | 140 |
| 加油 | jiāyóu | oil; fuel charging; make a greater effort | 124 |
| 东西 | dōngxī | thing | 123 |
| 咖啡 | kāfēi | coffee | 120 |
| 体验 | tǐyàn | experience; inspect | 120 |
| 图片 | túpiàn | picture; photograph | 115 |
| 夜店 | yèdiàn | nightclub | 114 |
| 赶紧 | gǎnjǐn | lose no time; hasten | 113 |
| 关注 | guānzhù | follow with interest; pay close attention to | 112 |
| 位置 | wèizhì | seat; place; location | 107 |
| 很多 | hěnduō | a lot of | 106 |
| 寻找 | xúnzhǎo | seek; look for; search | 106 |
| 居然 | jūrán | unexpectedly; to one's surprise | 104 |
| 周围 | zhōuwéi | around; round; about | 99 |
| 最后 | zuìhòu | last; final; ultimate | 95 |
| 勋章 | xūnzhāng | medal; decoration | 91 |
| 其实 | qíshí | actually; in fact; as a matter of fact | 86 |
| 孩子 | háizi | child | 85 |
| 妈妈 | māma | mom; mum | 78 |
| 投票 | tóupiào | vote; cast a vote | 77 |
| 排名 | páimíng | rank | 76 |
| 工作 | gōngzuò | work; job | 76 |
| 任务 | rènwu | assignment; mission; task | 75 |
| 老师 | lǎoshī | teacher | 74 |
| 无聊 | wúliáo | bored; in extreme depression | 73 |
| 永远 | yǒngyuǎn | forever; always | 71 |
| 挑战 | tiǎozhàn | challenge | 67 |
| 讨厌 | tǎoyàn | disagreeable; disgusting | 66 |
| 选择 | xuǎnzé | choice; choose; select | 64 |
| 继续 | jìxù | continue; go on | 64 |
| 帮助 | bāngzhù | help; assistance; assist | 63 |
| 麻将 | májiàng | mahjong | 61 |
| 传说 | chuánshuō | legend; it is said | 60 |
| 问题 | wèntí | question; problem | 60 |
| 告诉 | gàosù | tell | 60 |
| 衣服 | yīfu | clothing; clothes; dress | 57 |
| 温暖 | wēnnuǎn | warm; warmth | 57 |
| 支持 | zhīchí | sustain; hold out; support | 54 |
| 奖励 | jiǎnglì | encourage and reward; award; reward | 53 |
| 同学 | tóngxué | schoolmate; classmate; comrade | 51 |
| 应该 | yīnggāi | should; ought to; must | 49 |
| 非常 | fēicháng | very; extraordinary; unusual | 49 |
| 期待 | qīdài | anticipate; await; expect | 48 |
| 准备 | zhǔnbèi | prepare; get ready | 48 |
| 复习 | fùxí | review; revise | 46 |
| 身边 | shēnbiān | at one's side | 45 |
| 呵呵 | hēhē | an onomatopoeic word for laughter | 44 |
| 等级 | děngjí | grade; rank; order and degree | 44 |
| 宝贝 | bǎobèi | treasure; baby | 43 |
| 评论 | pínglùn | comment on; discuss | 42 |
| 胜利 | shènglì | win; victory; triumph | 42 |
| 火车 | huǒchē | train | 41 |
| 改变 | gǎibiàn | change; alter; transform | 40 |
| 寂寞 | jìmò | lonely; lonesome | 40 |
| *曼联 | mànlián | Manchester United | 40 |
| 意思 | yìsi | meaning | 40 |
| 休息 | xiūxī | rest; break; have a rest | 40 |
| 青春 | qīngchūn | youth | 40 |
| *杨幂 | yángmì | someone’s name | 39 |
| 摩羯 | mójié | Capricorn | 38 |
| 辛苦 | xīnkǔ | hard; toilsome | 38 |
| *北京 | běijīng | capital city of China | 37 |
| 爸爸 | bàba | papa; dad | 37 |
| 视频 | shìpín | video | 37 |
| 公司 | gōngsī | company; corporation; firm | 36 |
| 扑克 | pūkè | poker | 36 |
| 放弃 | fàngqì | give up; abandon | 35 |
| 父母 | fùmǔ | parent | 35 |
| 精神 | jīngshén | spirit; mind; consciousness | 35 |
| 保佑 | bǎoyòu | bless | 34 |
| 算算 | suànsuan | calculate | 34 |
| 妹妹 | mèimèi | younger sister | 33 |
| 免费 | miǎnfèi | free of charge; cost free; free | 32 |
| 健康 | jiànkāng | health; healthy; wholesome | 32 |
| 漂亮 | piàoliang | pretty; beautiful; good-looking | 32 |
| 羡慕 | xiànmù | admire; envy | 31 |
| 简单 | jiǎndān | simple; uncomplicated; briefness | 30 |
| 粉丝 | fěnsī | vermicelli made from bean starch; a slang meaning "fans" | 30 |
| 金币 | jīnbì | gold coin | 29 |
| 英雄 | yīngxióng | hero | 29 |
| 轻松 | qīngsōng | light; relaxed | 29 |
| 即使 | jíshǐ | even; even if; even though | 28 |
| 礼物 | lǐwù | gift; present | 28 |
| 宿舍 | sùshè | living quarters; dorm | 28 |
| 适合 | shìhé | suit; fit; be appropriate for | 27 |
| *广州 | guǎngzhōu | a city of China | 26 |
| 嘿嘿 | hēihēi | an onomatopoeic word for laughter | 26 |
| 密码 | mìmǎ | cipher; code | 26 |
| 疯狂 | fēngkuáng | insane; frenzied | 26 |
| 认识 | rènshí | know; be familiar with; be acquainted with | 26 |
| 演唱 | yǎnchàng | sing (in a performance) | 26 |
| 阳光 | yángguāng | sunshine | 26 |
| 高兴 | gāoxìng | happy; glad | 25 |
| 兄弟 | xiōngdì | brothers | 24 |
| 圣诞 | shèngdàn | Christmas | 23 |
| 姐姐 | jiějiě | elder sister | 23 |
| 姑娘 | gūniang | girl | 23 |
| 必须 | bìxū | must; necessary; have to | 23 |
| 往往 | wǎngwǎng | often; frequently; sometimes | 23 |
| 尼玛 | nímǎ | a Tibetan name; a slang swear word | 22 |
| 蛋糕 | dàngāo | cake | 22 |
| 清楚 | qīngchǔ | clear; distinct; lucid | 22 |
| 洗澡 | xǐzǎo | have a bath; bathe | 22 |
| 专业 | zhuānyè | specialized subject; speciality | 22 |
| 状态 | zhuàngtài | state; status; condition | 22 |
| 甜蜜 | tiánmì | sweet; happy | 22 |
| 嘻嘻 | xīxī | an onomatopoeic word for laughter | 21 |
| 医院 | yīyuàn | hospital | 21 |
| 减肥 | jiǎnféi | lose weight | 21 |
| 遗憾 | yíhàn | regret; pity; regretful; sorry | 21 |
| 逛街 | guàngjiē | saunter; stroll along the street | 20 |
| 烦恼 | fánnǎo | be vexed; be worried | 19 |
| 黑色 | hēisè | black | 19 |
| 矛盾 | máodùn | contradiction; contradict | 19 |
| 满足 | mǎnzú | satisfy; satisfied; content | 19 |
| 目标 | mùbiāo | target; objective; goal; aim | 19 |
| 千万 | qiānwàn | ten million; be sure | 19 |
| 呜呜 | wūwū | an onomatopoeic word | 18 |
| 申请 | shēnqǐng | application; apply; apply for | 18 |
| 政府 | zhèngfǔ | government | 18 |
| 历史 | lìshǐ | history; past records | 18 |
| 坑爹 | kēngdiē | a slang meaning "cheating" or "deceiving" | 17 |
| 裁判 | cáipàn | judgment; referee; judge | 17 |
| 慢慢 | mànmàn | slowly; gradually | 17 |
| 研究 | yánjiū | study; research | 17 |
| 哥哥 | gēge | elder brother | 16 |
| 建造 | jiànzào | formation; construct; build | 16 |
| *香港 | xiānggǎng | Hong Kong | 16 |
| 默默 | mòmò | silently; quietly | 16 |
| 欣赏 | xīnshǎng | appreciate; enjoy; admire | 15 |
| 艺术 | yìshù | art | 15 |
| 冠军 | guànjūn | champion; gold medalist | 15 |
| 便宜 | piányi | cheap; inexpensive | 15 |
| 爷爷 | yéye | grandpa | 15 |
| 团购 | tuángòu | group buying | 15 |
| 屋企 | wūqǐ | a Cantonese word for "home" | 14 |
| 左右 | zuǒyòu | about; around; control | 14 |
| 恐惧 | kǒngjù | fear; dread; be afraid of | 14 |
| 瞧瞧 | qiáoqiáo | have a look; look | 14 |
| 资料 | zīliào | means; data; material | 14 |
| 干嘛 | gànma | why; what to do | 14 |
| 哎呀 | āiyā | an interjection | 13 |
| 控制 | kòngzhì | control; dominate; regulate | 13 |
| 杯具 | bēijù | cup; a slang meaning "tragedy" | 13 |
| 奶奶 | nǎinai | grandma | 13 |
| 虾米 | xiāmǐ | peeled, dried sea shrimp; small shrimp | 13 |
| 奔波 | bēnbō | rush about; be busy running about | 13 |
| 草菇 | cǎogū | straw mushroom | 13 |
| 兵器 | bīngqì | weaponry; weapons; arms | 13 |
| 降临 | jiànglín | befall; arrive; come | 12 |
| 舞蹈 | wǔdǎo | dance | 12 |
| 魔鬼 | móguǐ | devil; demon; monster | 12 |
| 委屈 | wěiqu | feel wronged; suffer from injustice | 12 |
| 双鱼 | shuāngyú | Pisces | 12 |
| 皮肤 | pífū | skin | 12 |
| 财富 | cáifù | wealth; fortune; riches | 12 |
| 童鞋 | tóngxié | baby shoes; a slang meaning "schoolmate" or "classmate" | 11 |
| 呼呼 | hūhū | an onomatopoeic word | 11 |
| 阿姨 | āyí | aunt | 11 |
| 宣布 | xuānbù | declare; proclaim | 11 |
| 环境 | huánjìng | environment; surroundings; circumstances | 11 |
| 介绍 | jièshào | introduce; present; recommend | 11 |
| 祈祷 | qídǎo | pray; say one’s prayers | 11 |
| 吵架 | chǎojià | quarrel; wrangle; have a row | 11 |
| 江湖 | jiānghú | rivers and lakes; complicated human world | 11 |
| 這樣 | zhèiyàng | so; such; like this; this way | 11 |
| *杰伦 | jiélún | someone’s name | 11 |
| 折磨 | zhémó | torment; rack; torture | 11 |
| 画壁 | huàbì | wall with murals | 11 |
| 終於 | zhōngyū | at last; in the end; finally | 10 |
| 娃娃 | wáwá | baby; child | 10 |
| 惨遭 | cǎnzāo | brutally | 10 |
| 欺负 | qīfù | bully; take advantage of | 10 |
| 诱惑 | yòuhuò | entice; tempt; seduce; lure | 10 |
| 幼稚 | yòuzhì | naïve | 10 |
| *貂蝉 | diāochán | name of a figure in Chinese history | 10 |
| 叔叔 | shūshū | uncle | 10 |
| 嗷嗷 | áoáo | an onomatopoeic word | 9 |
| 巨蟹 | jùxiè | Cancer | 9 |
| 狗狗 | gǒugǒu | dog | 9 |
| 葡萄 | pútáo | grape | 9 |
| 障碍 | zhàng’ài | obstacle; hinder; obstruct | 9 |
| 土豆 | tǔdòu | potato | 9 |
| 迷茫 | mímáng | vast and hazy | 9 |
| 嘟嘟 | dūdū | an onomatopoeic word | 8 |
| 观众 | guānzhòng | audience | 8 |
| 崩溃 | bēngkuì | breakdown; collapse; crash | 8 |
| 蝴蝶 | húdié | butterfly | 8 |
| 宇宙 | yǔzhòu | cosmos; universe | 8 |
| 耳朵 | ěrduo | ear | 8 |
| 垃圾 | lājī | garbage; rubbish; trash | 8 |
| 猜猜 | cāicāi | guess | 8 |
| *库巴 | kùbā | name of a website | 8 |
| 骄傲 | jiāoào | proud; arrogant; conceited | 8 |
| 玫瑰 | méiguī | rose | 8 |
| 喉咙 | hóulóng | throat; the gullet | 8 |
| 疲惫 | píbèi | tired out; weary; become fagged; exhausted | 8 |
| 媳妇 | xífu | wife | 8 |
| 智慧 | zhìhuì | wisdom; intelligence | 8 |
| 嫉妒 | jídù | be jealous of; envy | 7 |
| 贡献 | gòngxiàn | contribution; contribute; dedicate | 7 |
| 嘉宾 | jiābīn | honoured guest | 7 |
| 痕迹 | hénjì | mark; imprint; vestige | 7 |
| *鹿鼎 | lùdǐng | name of a mountain | 7 |
| 营养 | yíngyǎng | nutrition; nourishment | 7 |
| 悄悄 | qiāoqiāo | quietly; on the quiet | 7 |
| 牙膏 | yágāo | toothpaste | 7 |
| 暧昧 | àimèi | ambiguous; equivocal; dubious | 6 |
| 嘤嘤 | yīngyīng | an onomatopoeic word | 6 |
| 哼哼 | hēnghēng | an onomatopoeic word | 6 |
| 慈善 | císhàn | charitable; benevolent; philanthropic | 6 |
| 辣椒 | làjiāo | chilli; hot pepper | 6 |
| 培训 | péixùn | cultivate; train | 6 |
| 朦胧 | ménglóng | dim; obscure | 6 |
| 颓废 | tuífèi | dispirited; decadent | 6 |
| 驾驶 | jiàshǐ | drive; pilot; steer | 6 |
| 虚拟 | xūnǐ | invented; fictitious; virtual | 6 |
| 糊涂 | hútu | muddled; confused; bewildered | 6 |
| 凤凰 | fènghuáng | phoenix | 6 |
| 萝卜 | luóbo | radish; turnip | 6 |
| 狠狠 | hěnhěn | ruthless; ferocious; firm | 6 |
| 偷偷 | tōutōu | stealthily; secretly | 6 |
| 趋势 | qūshì | trend; tendency; current | 6 |
| 沧桑 | cāngsāng | vicissitude; great changes | 6 |
| 乖乖 | guāiguāi | well-behaved; obedient | 6 |
| 吼吼 | hǒuhǒu | a slang onomatopoeic word for laughter | 5 |
| 嘎嘎 | gāgā | an onomatopoeic word; great | 5 |
| 搭配 | dāpèi | assort or arrange in pairs or groups; collocation | 5 |
| 召唤 | zhàohuàn | call; summon | 5 |
| 電腦 | diànnǎo | computer | 5 |
| 偏偏 | piānpiān | deliberately; just; only | 5 |
| 饮茶 | yǐnchá | drink tea | 5 |
| 脂肪 | zhīfáng | fat; axunge | 5 |
| 渐渐 | jiànjiàn | gradually; by degrees; little by little | 5 |
| 犹豫 | yóuyù | hesitate; be irresolute | 5 |
| 凌晨 | língchén | in the small hours; before dawn | 5 |
| 闲暇 | xiánxiá | leisure | 5 |
| 旋律 | xuánlǜ | melody | 5 |
| 舅舅 | jiùjiù | mother's brother | 5 |
| 潇洒 | xiāosǎ | natural and unrestrained | 5 |
| 洋洋 | yángyáng | numerous; copious | 5 |
| 诡异 | guǐyì | strange | 5 |
| 皱纹 | zhòuwén | wrinkle; lines; furrow | 5 |
| 唧唧 | jījī | an onomatopoeic word | 4 |
| 揭谛 | jiēdì | Chinese transliteration of a Sanskrit word | 4 |
| 咨询 | zīxún | consult; seek advice from | 4 |
| 含蓄 | hánxù | contain; embody; implict | 4 |
| 僵尸 | jiāngshī | corpse | 4 |
| 诅咒 | zǔzhòu | curse; swear; wish sb. evil | 4 |
| 侦探 | zhēntàn | detective | 4 |
| 挖掘 | wājué | dig; tap; excavate | 4 |
| 討厭 | tǎoyàn | disagreeable; disgusting | 4 |
| 浏览 | liúlǎn | glance over; skim through; browse | 4 |
| 匆匆 | cōngcōng | hurriedly; in a rush | 4 |
| 愤怒 | fènnù | indignation; anger; wrath; rage | 4 |
| 钥匙 | yàoshi | key | 4 |
| 悠悠 | yōuyōu | long; long-drawn-out; remote | 4 |
| 疙瘩 | gēda | lump; pimple; knot | 4 |
| 媽媽 | māma | mom; mum | 4 |
| 忐忑 | tǎntè | perturbed; mentally disturbed | 4 |
| 轉發 | zhuǎnfā | retransmission; transmit; relay | 4 |
| 牺牲 | xīshēng | sacrifice | 4 |
| 卧铺 | wòpù | sleeper; couchette | 4 |
| *范范 | fànfàn | someone’s name | 4 |
| 锻炼 | duànliàn | take exercise; have physical training | 4 |
| 尿尿 | niàoniào | urinate | 4 |
| 饕餮 | tāotiè | a mythical ferocious animal | 3 |
| 痘痘 | dòudòu | acne | 3 |
| 啪啪 | pāpā | an onomatopoeic word | 3 |
| 岩岩 | yányán | another form of 啱啱 ("just" in Cantonese) | 3 |
| 蝙蝠 | biānfú | bat | 3 |
| 唠叨 | láodao | chatter; garrulous | 3 |
| 悬崖 | xuányá | cliff; precipice | 3 |
| 連續 | liánxù | continuing; running; continuous; successive | 3 |
| 咳嗽 | késou | cough | 3 |
| 沮丧 | jǔsàng | dispirited; depressed; dejected | 3 |
| 狐狸 | húli | fox | 3 |
| 虫虫 | chóngchóng | insect; worm | 3 |
| 翡翠 | fěicuì | jadeite | 3 |
| 剛剛 | gānggāng | just; only; just now | 3 |
| 漏洞 | lòudòng | leak; flaw | 3 |
| 憧憬 | chōngjǐng | long for; look forward to | 3 |
| 瞅瞅 | chǒuchǒu | look; look at | 3 |
| *秦淮 | qínhuái | name of a river | 3 |
| 歪歪 | wāiwāi | oblique; crooked; name of a video-based social media platform | 3 |
| 準備 | zhǔnbèi | prepare; get ready | 3 |
| 惩罚 | chéngfá | punish; penalize; punishment | 3 |
| 寺庙 | sìmiào | temple | 3 |
| 馄饨 | húntún | wonton | 3 |
| 啱啱 | yányán | a Cantonese word meaning "just" | 2 |
| 蜀黍 | shǔshǔ | a slang meaning "uncle" | 2 |
| 呱呱 | gūgū | an onomatopoeic word | 2 |
| 喀嚓 | kāchā | an onomatopoeic word | 2 |
| 葫芦 | húlu | bottle gourd | 2 |
| 璀璨 | cuǐcàn | bright; resplendent | 2 |
| 烹饪 | pēngrèn | cooking; culinary art | 2 |
| 珊瑚 | shānhú | coral | 2 |
| 翩翩 | piānpiān | dance lightly; gracefully moving | 2 |
| 惆怅 | chóuchàng | disconsolate; melancholy | 2 |
| 選舉 | xuànjǔ | elect; election; vote | 2 |
| 玻璃 | bōlí | glass | 2 |
| 鬱悶 | yùmēn | gloomy; depressed | 2 |
| 習慣 | xíguàn | habit; custom; be used to | 2 |
| 憔悴 | qiáocuì | haggard; wan and sallow | 2 |
| 篱笆 | líba | hedge | 2 |
| 徘徊 | páihuái | hover; linger about | 2 |
| 聯賽 | liánsài | league matches | 2 |
| *虞姬 | yújī | name of a figure in Chinese history | 2 |
| 涅槃 | nièpán | nirvana | 2 |
| 猥琐 | wěisuǒ | of wretched appearance; of dreadful appearance | 2 |
| 團體 | tuán體 | organization; group; team | 2 |
| 兜兜 | dōudōu | pocket; move round | 2 |
| 讀書 | dúshū | read; study; attend school | 2 |
| 盗墓 | dàomù | rob a tomb; rob a grave | 2 |
| 應該 | yìnggāi | should; ought to; must | 2 |
| *旺旺 | wàngwàng | someone’s name | 2 |
| *贤茜 | xiánqiàn | someone’s name | 2 |
| *函函 | hánhán | someone’s name | 2 |
| 愚蠢 | yúchǔn | stupid; foolish; silly | 2 |
| 啰嗦 | luōsuō | talkative; long-winded | 2 |
| 覆辙 | fùzhé | the track of an overturned cart | 2 |
| 荆棘 | jīngjí | thistles and thorns | 2 |
| 寶貝 | bǎobèi | treasure; baby | 2 |
| 涓涓 | juānjuān | trickling sluggishly | 2 |
| 戰爭 | zhànzhēng | war; warfare | 2 |

Table C2. Word-like chunks (9 in total)

| **TNI** | **Phonetic transcription** | **English gloss** | **Height** |
| --- | --- | --- | --- |
| 我的 | wǒde | my | 469 |
| 靠靠 | kàokào | duplicated form of 靠 (a swear word) | 139 |
| 啦啦 | lālā | duplicated form of 啦 (an interjection) | 32 |
| 揉腹 | róufù | knead the belly | 4 |
| 雷雷 | léiléi | duplicated form of 雷 (a slang meaning ‘shocking’) | 3 |
| 耶耶 | yēyē | duplicated form of 耶 (an interjection) | 3 |
| 湘湘 | xiāngxiāng | part of someone’s screen name | 3 |
| 囧囧 | jiǒngjiǒng | duplicated form of 囧 (a slang meaning ‘embarrassed’) | 2 |
| 溜溜 | liūliū | a part of words such as 滑溜溜 (huáliūliū, ‘slippery’) and 灰溜溜 (huīliūliū, ‘gloomy’ or ‘dejected’) | 2 |

**Appendix D: Two-Node islands (TNIs) extracted from three sub-networks of Network LCMC_J**

Table D1. Two-character words extracted from Network LCMC_J_1 (17 in total, with those not extracted from Network LCMC_J underlined)

| **TNI** | **Phonetic transcription** | **English gloss** | **Height** |
| --- | --- | --- | --- |
| 劳动 | láodòng | work; labor; physical labor | 20 |
| 技术 | jìshù | technology; skill | 5 |
| 作用 | zuòyòng | effect; function | 4 |
| 支配 | zhīpèi | dominate; control; arrange | 3 |
| 时代 | shídài | era; times; age; epoch | 3 |
| 提高 | tígāo | increase; raise; enhance | 3 |
| 体力 | tǐlì | physical strength; physical capacity | 3 |
| 尽管 | jìnguǎn | despite; although | 2 |
| 因素 | yīnsù | factor; element | 2 |
| 简单 | jiǎndān | simple; uncomplicated; briefness | 2 |
| 直接 | zhíjiē | direct; straight; immediate | 2 |
| 包括 | bāokuò | include; consist of; comprise | 2 |
| 数量 | shùliàng | quantity; number; amount; quantum | 2 |
| 强大 | qiángdà | powerful; formidable; mightiness | 2 |
| 本身 | běnshēn | oneself; in itself; per se | 2 |
| 相比 | xiāngbǐ | compare with | 2 |
| 之间 | zhījiān | between | 2 |

Table D2. Word-like chunks extracted from Network LCMC_J_1 (2 in total)

| **TNI** | **Phonetic transcription** | **English gloss** | **Height** |
| --- | --- | --- | --- |
| 较少 | jiàoshǎo | less | 2 |
| 也对 | yěduì | also (have an effect) on | 2 |

Table D3. Two-character words extracted from Network LCMC_J_2 (37 in total, with those not extracted from Network LCMC_J underlined)

| **TNI** | **Phonetic transcription** | **English gloss** | **Height** |
| --- | --- | --- | --- |
| 电脑 | diànnǎo | computer | 33 |
| 银行 | yínháng | bank | 22 |
| 处理 | chùlǐ | handle; manage; deal with | 13 |
| 业务 | yèwù | business; service; operation | 10 |
| 自动 | zìdòng | automatic; voluntarily; spontaneous | 7 |
| 通过 | tōngguò | via; pass through; by means of | 7 |
| 可以 | kěyǐ | can; may | 6 |
| 数据 | shùjù | data; record | 6 |
| 顾客 | gùkè | customer; client; shopper | 6 |
| 日常 | rìcháng | daily; everyday; usual | 6 |
| 出纳 | chū’nà | cashier; receive and pay out money or bills | 5 |
| 信息 | xìnxī | information; message; news | 5 |
| 工作 | gōngzuò | work; job | 5 |
| 只要 | zhǐyào | provided; so long as; as long as | 5 |
| 转账 | zhuǎnzhàng | transfer accounts | 5 |
| 卡片 | kǎpiàn | card | 5 |
| 计算 | jìsuàn | calculate; compute; count | 4 |
| 发展 | fāzhǎn | develop; growth | 4 |
| 中央 | zhōngyāng | card; fiche | 3 |
| 准确 | zhǔnquè | accuracy; exact; precise | 3 |
| 磁盘 | cípán | magnetic disk | 3 |
| 差错 | chàcuò | error; mistake | 3 |
| 存款 | cúnkuǎn | deposit; savings | 3 |
| 阶段 | jiēduàn | stage; phase; period | 3 |
| 汇兑 | huìduì | remittance; transfer; exchange (in commerce) | 2 |
| 商店 | shāngdiàn | store; shop | 2 |
| 本人 | běnrén | me; oneself; self | 2 |
| 同时 | tóngshí | simultaneously; meanwhile; at the same time | 2 |
| 保证 | bǎozhèng | guarantee; ensure; assure | 2 |
| 技术 | jìshù | technology; skill | 2 |
| 按键 | ànjiàn | key; push-button | 2 |
| 故障 | gùzhàng | fault; breakdown | 2 |
| 许多 | xǔduō | many; much | 2 |
| 预测 | yùcè | forecast; calculate | 2 |
| 随着 | suízhe | along with; in the wake of; in pace with | 2 |
| 落后 | luòhòu | backward; fall behind | 2 |
| 签字 | qiānzì | sign; affix one's signature | 2 |

Table D4. Word-like chunks extracted from Network LCMC_J_2 (3 in total)

| **TNI** | **Phonetic transcription** | **English gloss** | **Height** |
| --- | --- | --- | --- |
| 狭缝 | xiáféng | narrow crack; slit | 4 |
| 一张 | yīzhāng | a piece (sheet) of | 3 |
| 很小 | hěnxiǎo | very small | 2 |

Table D5. Two-character words extracted from Network LCMC_J_3 (65 in total, with those not extracted from Network LCMC_J underlined)

| **TNI** | **Phonetic transcription** | **English gloss** | **Height** |
| --- | --- | --- | --- |
| 肥胖 | féipàng | fat; obesity | 70 |
| 脂肪 | zhīfáng | fat; axunge | 60 |
| 胰岛 | yídǎo | pancreatic islets | 29 |
| 细胞 | xìbāo | cell | 29 |
| 分泌 | fènmì | secrete; secretion | 21 |
| 正常 | zhèngcháng | normal; regular | 17 |
| 组织 | zǔzhī | organization; organize; tissue | 16 |
| 神经 | shénjīng | nerve; nervous | 13 |
| 合成 | héchéng | compose; compound; synthetize | 13 |
| 皮质 | pízhì | cortex | 11 |
| 食欲 | shíyù | appetite; belly; orexia | 10 |
| 作用 | zuòyòng | effect; function | 10 |
| 功能 | gōngnéng | function | 10 |
| 代谢 | dàixiè | supersession; metabolize | 10 |
| 主要 | zhǔyào | main; major | 9 |
| 发生 | fāshēng | occur; occurrence; happen | 9 |
| 血糖 | xuètáng | blood sugar | 9 |
| 存在 | cúnzài | exist; existence | 8 |
| 活动 | huódòng | activity; campaign; exercise | 8 |
| 遗传 | yíchuán | inheritance; heredity; inherit | 7 |
| 改变 | gǎibiàn | change; alter; transform | 7 |
| 中枢 | zhōngshū | center; backbone | 7 |
| 影响 | yǐngxiǎng | influence; impact; affect | 7 |
| 体重 | tǐzhòng | (body) weight | 7 |
| 调节 | tiáojié | adjust; regulate; monitor | 6 |
| 认为 | rènwéi | think; consider; deem | 6 |
| 水平 | shuǐpíng | level; horizontal; standard | 6 |
| 促进 | cùjìn | promote; boost; advance | 6 |
| 增多 | zēngduō | increase; grow in number or quantity | 6 |
| 抑制 | yìzhì | restrain; control; inhibition | 5 |
| 引起 | yǐnqǐ | cause; give rise to; lead to | 5 |
| 一般 | yībān | general; ordinary; common | 5 |
| 习惯 | xíguàn | habit; custom; be used to | 5 |
| 明显 | míngxiǎn | obvious; clear; evident | 5 |
| 子女 | zǐnǚ | children; offspring | 5 |
| 升高 | shēnggāo | rise; go up | 5 |
| 减少 | jiǎnshǎo | reduce; decrease; lessen | 4 |
| 消耗 | xiāohào | consume; use u | 4 |
| 营养 | yíngyǎng | nutrition; nourishment | 4 |
| 由于 | yóuyú | thanks to; as a result of; due to | 4 |
| 不同 | bùtóng | difference; distinct | 4 |
| 通过 | tōngguò | via; pass through; by means of | 4 |
| 基础 | jīchǔ | foundation; basis; base | 4 |
| 环境 | huánjìng | environment; surroundings; circumstances | 4 |
| 部位 | bùwèi | part; location; place | 4 |
| 紊乱 | wěnluàn | disorder; chaos; confusion | 4 |
| 有关 | yǒuguān | have something to do with; relate to; concern | 3 |
| 差别 | chābié | difference; distinction; disparity | 3 |
| 其他 | qítā | other; else; rest | 3 |
| 以后 | yǐhòu | after; later; afterwards | 3 |
| 降低 | jiàngdī | reduce; cut down | 3 |
| 相互 | xiānghù | mutual; reciprocal; each other | 3 |
| 容易 | róngyì | easily; easy; likely | 3 |
| 饥饿 | jī’è | hunger; starvation; famine | 3 |
| 反应 | fǎnyìng | reaction; response | 3 |
| 双亲 | shuāngqīn | (both) parents | 3 |
| 抵抗 | dǐkàng | resistance; resist; stand up to | 3 |
| 纤维 | xiānwéi | fiber; staple; filamentary | 2 |
| 方面 | fāngmiàn | aspect; side | 2 |
| 随着 | suízhe | along with; in the wake of; in pace with | 2 |
| 往往 | wǎngwǎng | often; frequently; sometimes | 2 |
| 休息 | xiūxī | rest; break; have a rest | 2 |
| 睾丸 | gāowán | testicle | 2 |
| 研究 | yánjiū | study; research | 2 |
| 之间 | zhījiān | between | 2 |

Table D6. Word-like chunks extracted from Network LCMC_J_3 (4 in total)

| **TNI** | **Phonetic transcription** | **English gloss** | **Height** |
| --- | --- | --- | --- |
| 微克 | wēikè | microgram | 6 |
| 儿期 | érqī | a part of word, as in 婴儿期 (yīngérqī, (earlier) infancy) and幼儿期 (yòuérqī, (later) infancy) | 4 |
| 侧核 | cèhé | lateral nucleus | 3 |
| 这种 | zhèzhǒng | this kind | 3 |
